# Functionally Significant Features in the 5′ Untranslated Region of the ABCA1 Gene and Their Comparison in Vertebrates

**DOI:** 10.3390/cells8060623

**Published:** 2019-06-21

**Authors:** Pavel Dvorak, Sarah Leupen, Pavel Soucek

**Affiliations:** 1 Department of Biology, Faculty of Medicine in Pilsen, Charles University, Alej Svobody 76, 32300 Pilsen, Czech Republic; 2 Biomedical Center, Faculty of Medicine in Pilsen, Charles University, Alej Svobody 76, 32300 Pilsen, Czech Republic; pavel.soucek@szu.cz; 3 Department of Biological Sciences, University of Maryland Baltimore County, Baltimore, MD 21250, USA; leupen@umbc.edu; 4 Toxicogenomics Unit, National Institute of Public Health, Srobarova 48, 100 42 Prague 10, Czech Republic

**Keywords:** 5′ untranslated region, gene regulation, single nucleotide polymorphism, ABCA1, bioinformatics

## Abstract

Single nucleotide polymorphisms located in 5′ untranslated regions (5′UTRs) can regulate gene expression and have clinical impact. Recognition of functionally significant sequences within 5′UTRs is crucial in next-generation sequencing applications. Furthermore, information about the behavior of 5′UTRs during gene evolution is scarce. Using the example of the ATP-binding cassette transporter A1 (*ABCA1*) gene (Tangier disease), we describe our algorithm for functionally significant sequence finding. 5′UTR features (upstream start and stop codons, open reading frames (ORFs), GC content, motifs, and secondary structures) were studied using freely available bioinformatics tools in 55 vertebrate orthologous genes obtained from Ensembl and UCSC. The most conserved sequences were suggested as hot spots. Exon and intron enhancers and silencers (sc35, ighg2 cgamma2, ctnt, gh-1, and fibronectin eda exon), transcription factors (TFIIA, TATA, NFAT1, NFAT4, and HOXA13), some of them cancer related, and microRNA (hsa-miR-4474-3p) were localized to these regions. An upstream ORF, overlapping with the main ORF in primates and possibly coding for a small bioactive peptide, was also detected. Moreover, we showed several features of 5′UTRs, such as GC content variation, hairpin structure conservation or 5′UTR segmentation, which are interesting from a phylogenetic point of view and can stimulate further evolutionary oriented research.

## 1. Introduction

The need to recognize functionally relevant sequences in specific genes becomes more important with the massive generation of data by various next-generation sequencing techniques in research as well as routine laboratory practice (in both whole exome as well as whole genome sequencing). It is known that single nucleotide polymorphisms (SNPs) located in untranslated regions (UTRs) can regulate gene expression; however, these regions are often ignored by whole exome sequencing approaches [1]. At the same time, there remains a lack of information about the behavior of gene subsections such as 5′ and 3′ untranslated regions (5′UTRs, 3′UTRs) during gene evolution. Research in these closely connected issues has the potential to discover new clinically important findings and is addressed within the current article.

Members of the ATP-binding cassette (ABC) gene family are classified into subfamilies based on similarities in gene structure and ATP-binding and transmembrane domains (seven subfamilies have been described in mammals). Proteins coded by ABC genes are mostly dedicated to transport functions across cell membranes, with an extraordinarily wide spectrum of substrates [2]. ABC transporters participate in diverse key cell processes such as membrane homeostasis, waste disposal and detoxification, cell signaling, lipid trafficking, drug resistance, and stem cell pluripotency [3]. However, other ABC proteins, which lack the transmembrane domain and do not participate in active transport, have been connected with processes such as translation initiation, ribosome biogenesis and recycling, and innate immunity [4].

The human ATP-binding cassette transporter A1 (ABCA1) is a 247-kDa membrane-associated protein, which plays a major role in high density lipoprotein (HDL) biosynthesis and cellular cholesterol homeostasis. The human *ABCA1* gene is 149 kb long, contains 50 exons and is widely expressed, particularly in macrophages, hepatocytes, and enterocytes [5]. Mutations in this gene have been linked to an autosomal recessive disorder called Tangier disease, which is characterized by low plasma HDL levels, deposition of cholesterol in tissue macrophages, and prevalent atherosclerosis [6].

Cells modulate gene expression by alternative approaches at multiple levels—from DNA transcription to chromatin modification—in time and location-specific manners. The regulation of protein amount at the level of translation is thought to be one of the crucial points of regulation. The main role of 5′UTRs is seen in the control of translation initiation [7,8]. Different features within 5′UTRs influence both the efficiency and factor requirements for ribosome recruitment to specific mRNAs [9,10]. A variety of translation regulatory elements have been described and located to the 5′UTRs—secondary and tertiary structures (pseudoknots, hairpins, and RNA G-quadruplexes), internal ribosomal entry sites (IRESs), upstream start codons (uATGs/uAUGs) and open reading frames (uORFs), binding sites for RNA-binding proteins (RBPs) as well as the Kozak consensus sequence surrounding start codons of main ORFs (sATGs and mORFs, respectively) [11,12,13]. The importance of 5′ and 3′UTRs in regulating gene expression is underlined by the finding that congenital as well as acquired mutations that alter these regions can lead to serious pathology in humans [14,15].

Approximately 35% and 10% of human 5′ and 3′UTRs, respectively, are annotated as harboring introns [16,17]. Recent findings indicate that introns in UTRs should be considered to be distinctive entities with specific regulatory functions. The presence or absence of a 5′UTR intron has been connected with significant consequences for mRNA nuclear export and cytoplasmic metabolism. In the 3′UTRs, introns can modulate protein expression via targeting the mRNA for degradation by nonsense-mediated decay [16,17,18]. In this work we address the hypothesis that the position of introns within 5′UTRs is not random and has functional consequences.

**Figure 1:**
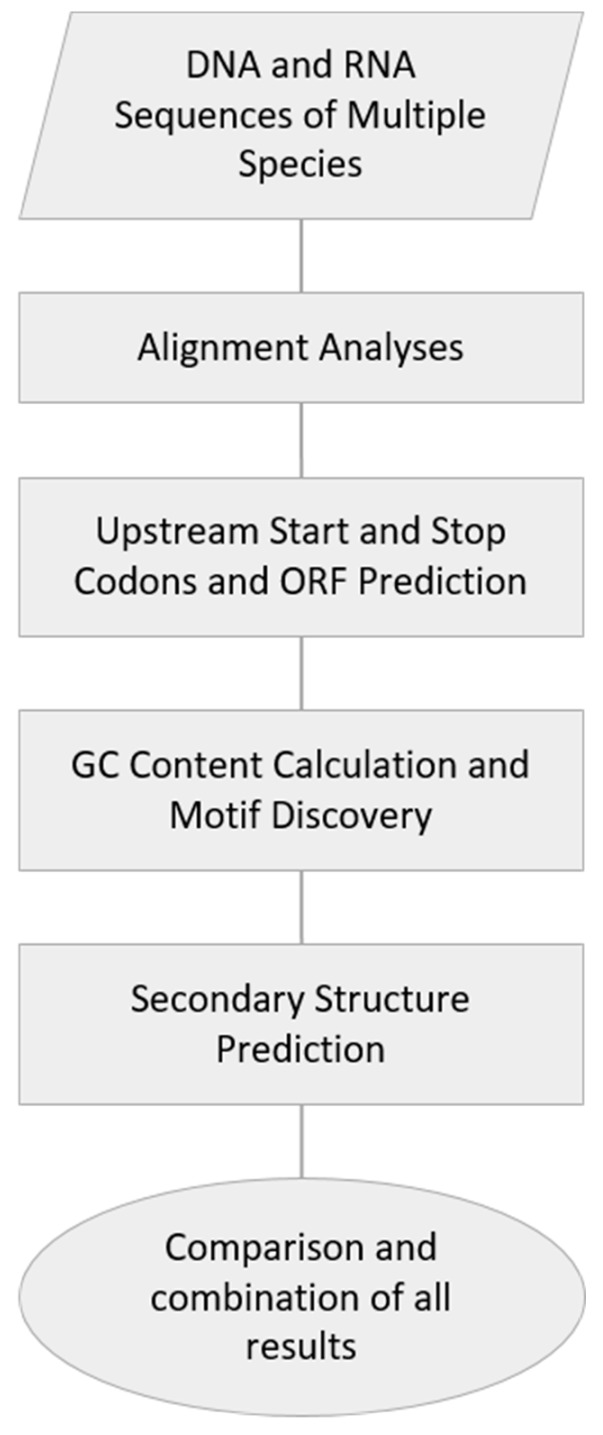
Workflow of significant sequence finding in 5′UTRs applied in this study. ORF, open reading frame.

Although a considerable body of evidence about 5′UTRs and their evolution is available in the literature, this knowledge is mainly based on the whole genome level approaches and analyses. Studies focusing on this theme in specific genes or gene families are still in the minority. ABC genes in particular constitute a large and clinically important family; however, precise information about the structure and evolution of their 5′UTRs is missing. The current study provides a comprehensive comparison of 5′UTRs in vertebrate *ABCA1* genes performed with the help of freely available bioinformatics tools. The algorithm of significant sequence characterization is, in general, applicable to any other gene of interest (Figure 1).

## 2. Materials and Methods

### 2.1. DNA and RNA Sequences of Multiple Species

**Figure 2:**
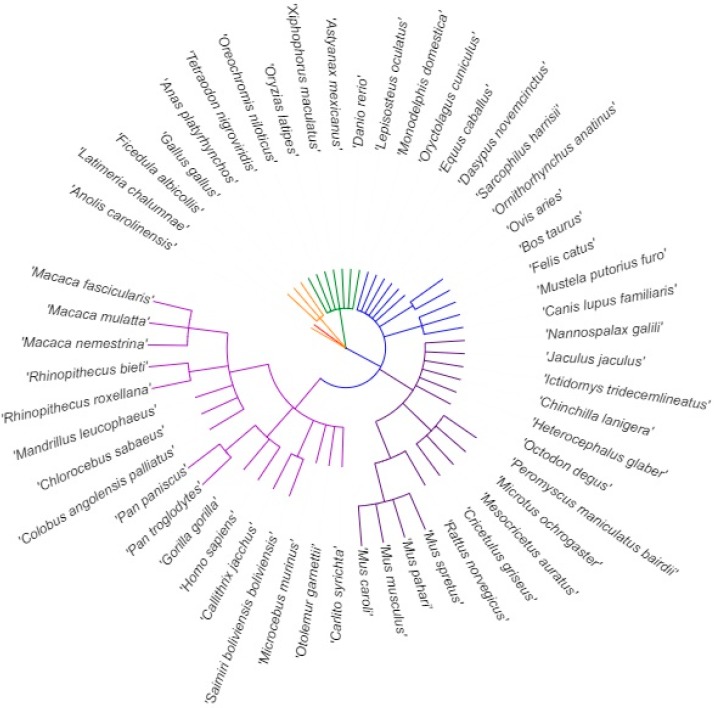
Simplified phylogenetic tree highlighting 55 vertebrate species evaluated in this study. Clades were colored as follows: Primates—light violet; Rodents—dark violet; Mammals—blue; Ray-finned fishes—green; Reptiles and birds—orange; and Coelacanth—red.

Searches for DNA sequences of genes orthologous to the human *ABCA1* gene were done in the Ensembl genome browser (https://www.ensembl.org/index.html; Ensembl release 91—December 2017, GRCh38.p10; accessed on 11 January 2018) [19]. In cases with more than one transcript available, the transcripts expected to code for the main functional isoform were selected based on the APPRIS system [20]. Two transcripts were retrieved for both naked mole-rat (*Heterocephalus glaber*)—naked mole-rat male and naked mole-rat female (based on the availability of these sequences in the database) and Tilapia (*Oreochromis niloticus*)—abca1a-201 and abca1b-201 (based on whole genome duplication in ray-finned fishes), and three transcripts for Zebrafish (*Danio rerio*)—abca1a-203, abca1b-201, and abca1b-202 (based on the uncertainty about the clear principal variant). All 5′UTR sequences without intron sequences—59 transcripts from 55 species—were downloaded in FASTA format and multi-FASTA format files containing 5′UTR sequences of all species studied and separately the main phylogenetic subgroups of vertebrates were created (Supplemental Table S1). Within these files, sequences were listed according to the known phylogenetic relations, which were adopted from the Gene Tree tool (developed and maintained by the Ensembl database, available at https://www.ensembl.org/index.html; accessed on 11 January 2018 and recorded in Supplemental Figure S1). Ensembl gene trees are generated by the Gene Orthology/Paralogy prediction method pipeline (description available at https://www.ensembl.org/info/genome/compara/homology_method.html) [21]. A simplified phylogenetic tree highlighting the vertebrate species, which were evaluated in this study, was prepared with the help of Taxonomy Browser (National Center for Biotechnology Information (NCBI), Bethesda MD, USA, accessed on 9 June 2019 at https://www.ncbi.nlm.nih.gov/Taxonomy/CommonTree/wwwcmt.cgi) and iTOL server (Interactive Tree Of Life (iTOL), European Molecular Biology Laboratory (EMBL), version 4.4.2, accessed on 9 June 2019 at https://itol.embl.de/), and is shown in Figure 2. Detailed information about the names and IDs of the species and transcripts evaluated was summarized in Supplemental Table S2.

The EMBOSS Transeq program (https://www.ebi.ac.uk/Tools/st/emboss_transeq/; accessed on 26 January 2018) was used to translate nucleic acid sequences to their corresponding peptide sequences where needed. 5′UTR DNA sequences were converted into RNA sequences using Reverse Transcription and Translation Tool (ExPASy-SIB Bioinformatics Resource Portal, Swiss Institute of Bioinformatics; https://www.expasy.org/; accessed on 29 January 2018).

### 2.2. Alignment Analyses

The comparative method has a long tradition in biology and has been naturally incorporated into the current bioinformatics approaches studying genomic sequences. Several types of alignment analyses are used to reveal conserved regions having key biological functions [22].

Multiple-sequence alignment analyses, covering all 59 transcripts downloaded from Ensembl, were performed with the help of Jalview software (open source project released under the GNU General Public License; University of Dundee, Dundee, UK; version Jalview 2.10.3; accessed on 25 January 2018; available at http://www.jalview.org/) [23] and Bioinformatics Tools available on the EMBL-EBI (Wellcome Genome Campus, Hinxton, UK) web pages (https://www.ebi.ac.uk/Tools/msa/; accessed on 25 January 2018). Results of the nine different alignment algorithms—T-Coffee, Probcons, Muscle, Mafft, MSAprobs, GLprobs, Clustal, ClustalO, and WebPRANK—were visualized in a similar form (alignment with percentages of nucleotide identity colored, histograms and logos of consensus sequences and histograms of occupancy values) and compared to each other in a precise comparative analysis. In parallel, results of the conservation track in the UCSC Genome Browser (https://genome-euro.ucsc.edu/index.html; accessed on 5 February 2018) [24], which shows multiple alignment results of 100 vertebrate species and measurements of evolutionary conservation using two methods (phastCons and phyloP) from the PHAST package (description at http://compgen.cshl.edu/phast/), were considered.

Pairwise sequence alignment was performed with the help of Bioinformatics Tools available on EMBL-EBI web pages (https://www.ebi.ac.uk/Tools/psa/; accessed on 6 February 2018); results of EMBOSS Water, Matcher, and Needle Tools were employed in the study.

### 2.3. Upstream Start and Stop Codons and ORF Prediction

Transcripts from 15 species were chosen as a representative sample set of vertebrate diversity—human, macaque, mouse lemur, squirrel, mouse, rabbit, cat, armadillo, Tasmanian devil, opossum, platypus, chicken, flycatcher, anole lizard, and coelacanth. Possible ORFs within ABCA1 gene transcripts were predicted with the help of two freely available web tools—ORFfinder (NCBI Resources, U.S. National Library of Medicine, Bethesda, Rockville, MD, USA; https://www.ncbi.nlm.nih.gov/orffinder/; accessed on 27 November 2018) and NetStart (DTU Bioinformatics, Technical University of Denmark, Kongens Lyngby, Denmark; version 1.0; http://www.cbs.dtu.dk/services/NetStart/; accessed on 27 November 2018). Compared to ORFfinder, NetStart has no minimal ORF length restriction and calculates translation start scores ranging between 0 and 1 for each possible ORF. On the other hand, alternative initiation start codons can be evaluated in ORFfinder. The human *ABCA1* cDNA sequence downloaded from Ensembl database was further evaluated for putative bioactive short peptides with the uPEPperoni program (University of Queensland, Brisbane, Australia; http://upep-scmb.biosci.uq.edu.au; accessed on 12 December 2018) [25]. The WeakAUG server (Department of Biological Sciences and Bioengineering, Indian Institute of Technology Kanpur, Kanpur, India, accessed on 18 December 2018 at http://bioinfo.iitk.ac.in/AUGPred/) implements a neural network method, which was developed to predict the initiation site of mRNA sequences that lack the preferred nucleotides at the positions −3 and +4 surrounding the translation initiation site [26].

### 2.4. GC Content Calculation and Motif Discovery

GC content was calculated as a percentage based on the formula: Count(G + C)/Count(A + T + G + C) × 100, following standard practice. Several freely available on-line calculators can be efficiently employed; we used the Genomics %G~C Content Calculator (Science Buddies, Sobrato Center for Nonprofits, Milpitas, CA, USA, https://www.sciencebuddies.org/science-fair-projects/references/genomics-g-c-content-calculator; accessed on 14 December 2018). GC Content Distribution Plots were obtained from GC Content Calculator (Biologics International Corp, Indianapolis, IN, USA, https://www.biologicscorp.com/tools/GCContent/index; accessed on 14 December 2018). Searches for UTR motifs located within the 5′UTRs of the 15 representative vertebrate species mentioned above were performed in several on-line tools independently. UTRscan pattern matcher (ITB CNR—Institute for Biomedical Technologies, Bari, Italy, version 2010, accessed on 11 May 2019, http://itbtools.ba.itb.cnr.it/utrscan) is able to find motifs that characterize 3′UTR and 5′UTR sequences. A collection of 58 functional sequence patterns located in the 5′- or 3′-UTR sequences is defined in the UTRSite Database (http://utrsite.ba.itb.cnr.it/index.php/default/; accessed on 11 May 2019) [27]. RegRNA 2.0 (Institute of Bioinformatics and System Biology, National Chiao Tung University, Taiwan, Release 2.0, Jun. 2012, accessed on 11 May 2019, http://regrna2.mbc.nctu.edu.tw/index.html) is an integrated web server for identifying functional RNA motifs and sites in an entered RNA sequence [28]. MEME Suite (University of Nevada, Reno, University of Washington, Seattle, WA, USA, Version 5.0.5; http://meme-suite.org/; accessed on 15 May 2019) allows biologists to discover novel motifs in collections of unaligned nucleotide or protein sequences, and to perform a wide variety of other motif-based analyses [29].

### 2.5. Secondary Structure Prediction

Secondary structures of 5′UTR RNA sequences were independently predicted by two web servers—RNAstructure (Mathews group, University of Rochester Medical Center, Rochester, NY, USA, http://rna.urmc.rochester.edu/RNAstructureWeb/; accessed on 30 November 2018) and RNAfold (Institute for Theoretical Chemistry, University of Vienna, Vienna, Austria, http://rna.tbi.univie.ac.at/cgi-bin/RNAWebSuite/RNAfold.cgi; accessed on 30 November 2018). The RNAstructure program—Predict a Secondary Structure Common to Three or More Sequences—combines Multilign (which predicts low free energy secondary structures common to three or more sequences using progressive iterations of Dynalign) and TurboFold (which calculates the conserved structures of three or more unaligned sequences using iteratively refined partition functions) algorithms. This program has a limit of 10 input sequences; for that reason, sequences of 10 species (human, mouse lemur, mouse, rabbit, armadillo, opossum, platypus, flycatcher, anole lizard, and coelacanth) covering important subgroups of vertebrates were selected for these analyses.

### 2.6. Statistics

Statistical analyses were conducted with the help of the freely available PAST statistical software package (Natural History Museum, University of Oslo, version 3.12, https://folk.uio.no/ohammer/past/).

## 3. Results

### 3.1. Length of 5′UTR Sections Located after-Intron-1 are Conserved within Main Vertebrate Subgroups

**Figure 3:**
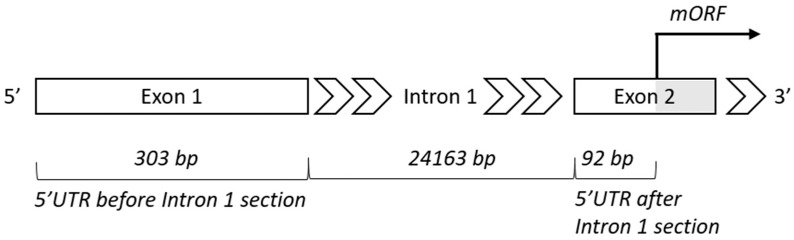
Detail of the 5′ end of the human *ABCA1* gene; 5′UTR, 5′ untranslated region; bp, base pairs; mORF, main open reading frame.

**Figure 4:**
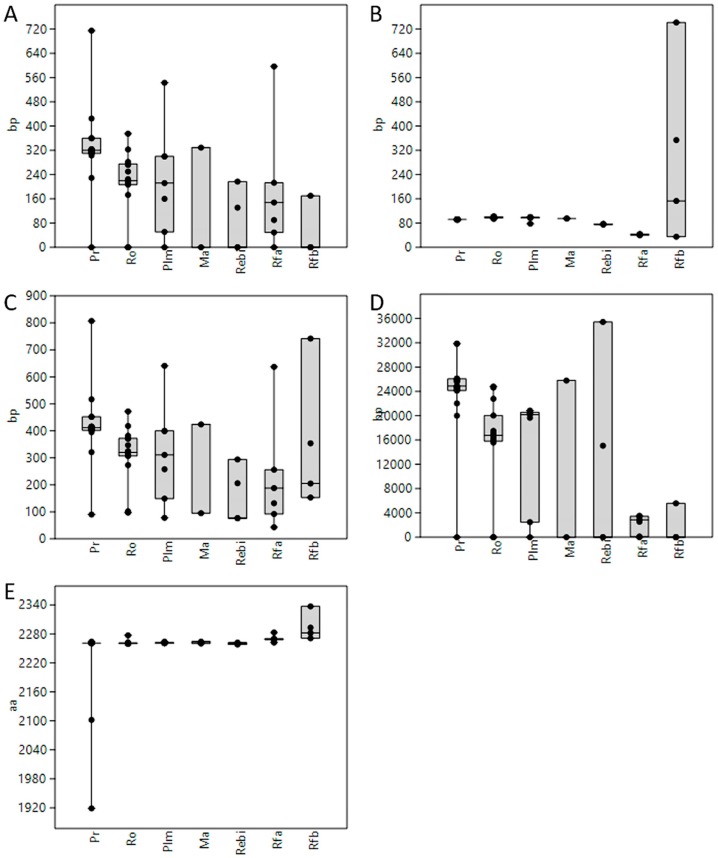
Length comparison of 5′UTRs, their subsections and ABCA1 proteins in vertebrates. (**A**) before-Intron-1 sections of 5′UTRs (Pr vs. Ro *p* = 0.018, Mann–Whitney pairwise, Bonferroni corrected *p* values); (**B**) after-Intron-1 sections (Pr vs. Ro *p* = 0.000003, Pr vs. Plm *p* = 0.031, Pr vs. Ma *p* = 0.012, Pr vs. Rebi *p* = 0.001, Pr vs. Rfa *p* = 0.0003); (**C**) whole 5′UTRs (Pr vs. Ro *p* = 0.032); (**D**) whole Intron 1 regions (Pr vs. Ro *p* = 0.004, Pr vs. Plm *p* = 0.043, Pr vs. Rfa *p* = 0.034); and (**E**) ABCA1 proteins (Pr vs. Rfa *p* = 0.002, Pr vs. Rfb *p* = 0.007). Pr., primates; Ro, rodents; Plm, other placental mammals; Ma, marsupials; Rebi, reptiles and birds; Rfa, ray-finned fishes–ABCA1a genes; Rfb, ray-finned fishes–ABCA1b genes.

DNA sequences of the *ABCA1* gene and its orthologues were available for 84 vertebrate species listed in the Ensembl database. In only 55 of these species—17 species of primates, 16 rodents, 7 other placental mammals, 2 marsupials, platypus (*Ornithorhynchus anatinus*), 4 reptiles and birds, coelacanth (*Latimeria chalumnae*), and 7 ray-finned fishes—5′UTR regions were annotated. Thus, 59 transcripts were selected for our analyses (see Materials and Methods for details). Twelve 5′UTRs (11 species—1 primate, 2 rodents, 1 other placental mammal, 1 marsupial, platypus, 2 reptiles and birds, and 3 ray-finned fishes) were not interrupted by any intron sequence. One intron sequence (named Intron 1 or Intron 1-2) was located within the rest of the 5′UTRs, i.e., 47 transcripts corresponding to 45 species including the most ancient ones—spotted gar (*Lepisosteus oculatus*) and coelacanth (*Latimeria chalumnae*). Figure 3 shows a detail of the 5′ end of human *ABCA1* gene schematically. Information about the lengths (in bp) of the following 5′UTR sections was collected: the whole 5′UTR region, sections before and after-Intron-1 and whole Intron 1 region (where relevant). Supplemental Table S2 discloses an overview of all the species covered, names and IDs of the transcripts downloaded and lengths of the 5′UTR sections evaluated. The lengths of all the 5′UTR sections mentioned above showed a decreasing trend in the direction primates, rodents, other placental mammals, marsupials, reptiles and birds, and ray-finned fishes, whereas the lengths of the final proteins (in aa) stayed constant. Interestingly, while the sections before-Intron-1 showed variability in length between species even within the main vertebrate subgroups, the length of the after-Intron-1 sections stayed mostly constant within these main subgroups, suggesting more important functional and evolutional roles of these 5′UTR sections (Figure 4).

### 3.2. 5′UTR Sections Located after-Intron-1 Start with Highly Conserved Sequences

**Figure 5:**
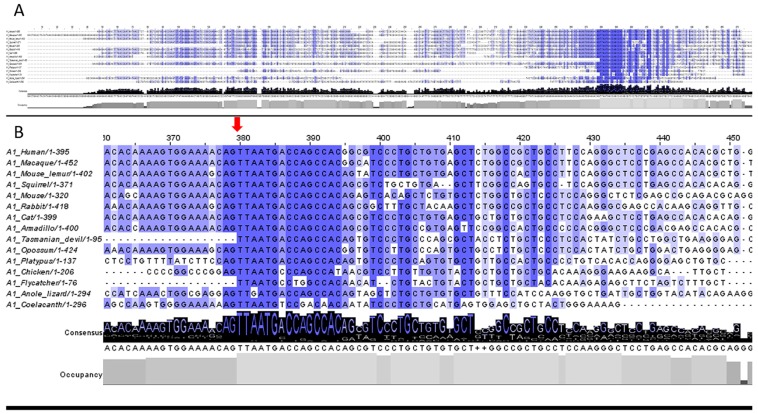
Results of the *ABCA1* 5′UTR multi-sequence alignment of transcripts from 15 vertebrate species performed in ClustalO and visualized in Jalview. Lines represent individual transcripts, the conservation of nucleotides among vertebrate species is highlighted with the most conserved ones having the darkest color, and levels of identity and occupancy scores are visible as histograms at the bottom. (**A**) Alignment of whole 5′UTR regions. 5′UTR segments, which contain conserved nucleotides, can be distinguished. (**B**) A detail of the 5′UTR segment, which contains the most conserved sequences, is provided. The position of the spliced Intron 1 is marked by a red arrow.

Multiple-sequence alignment analyses performed on the 59 transcripts downloaded from the Ensembl database were conducted with the help of nine different alignment programs (see Materials and Methods for details). The most conserved nucleotides (nts) within our data were defined as those having more than an 80% identity score and more than an 84% occupancy score (50 out of 59 sequences in minimum). Results of the alignment analyses indicated that there was a median of 37 (range 16–41) most conserved nts within the 5′UTRs evaluated. Importantly, almost all of these nts were concentrated within the after-Intron-1 sections. Furthermore, the longest continuous sequence composed of the most conserved nts was 15 nts long and localized at the start of the after-Intron-1 sections. Alignment results of transcripts from 15 selected vertebrate species performed in ClustalO program are shown in Figure 5; results of all 59 transcripts from 55 species are visualized in Supplemental Figure S2 and summarized in Supplemental Table S3.

Results of the UCSC conservation track (based on 100 vertebrate species) indicated three comparably long highly conserved regions (HCR1-HCR3) within the whole 5′UTR of the human *ABCA1* gene (with lod scores comparable to the lod scores of Exon 2 protein-coding sequences). HCR1 and 2 were assigned to the start of the 5′UTR before-Intron-1 section and HCR3 to the start of the after-Intron-1 section. HCR3 overlapped with the 15-nts highly conserved sequence revealed by the abovementioned alignment analyses (see Supplemental Figure S3 for details including lod scores).

### 3.3. Upstream ATG is Localized within the Highly Conserved Sequence at the Start of the 5′UTR after-Intron-1 Section

Analyses focusing on the number and positon of uATGs, non-ATG upstream start codons (uGTG, uCTG, uTTG, and uACG) as well as upstream stop codons (uTAG, uTAA, and uTGA) were carried out on the 59 transcripts downloaded from the Ensembl database. With a few exceptions (e.g., cow had 10 uATGs), the vast majority of species from primates to coelacanth had only one uATG (see Supplemental Table S4 for details). There was zero or one uATG present within the 5′UTR of ABCA1a genes in ray-finned fishes with the exception of the spotted gar who had five uATGs. The number of uATGs ranged between zero and seven in case of ABCA1b genes in ray-finned fishes. Importantly, it was recognized that the uATG, traceable from primates to coelacanth, was localized within the 15-nts highly conserved sequence at the start of the after-Intron-1 section (stressed by the alignment analyses). This out-of-frame uATG is located at position 307 from the transcription start site (TSS) within the human *ABCA1* gene.

The median number of uGTGs ranged between 2 and 6 with a decreasing trend from primates to ray-finned fishes (in ABCA1a genes). The distribution of uGTGs was similar in both 5′UTR sections—the before-Intron-1 and after-Intron-1 sections. The median number of uCTGs ranged between 3 in ray-finned fishes (ABCA1a genes) and 10 in primates. Starting from coelacanth to primates the incidence of uCTGs was higher within the after-Intron-1 sections than the before-Intron-1 sections (Supplemental Table S4 and Supplemental Figure S4). The median number of uTTGs ranged between 1 in marsupials and platypus to 7 in ray-finned fishes (in ABCA1b genes). In contrast to uCTGs, the incidence of uTTGs was higher within the before-Intron-1 sections (Supplemental Figure S5). The median number of uACGs ranged between 0 (coelacanth, reptiles and birds, and platypus and marsupials) and 4 (placental mammals), with no clear difference between the two sections. The median number of uTAGs ranged between 0 (coelacanth and reptiles and birds) and 3 (mammals and ABCA1b genes of ray-finned fishes). With a few exceptions, there were no uTAGs within the after-Intron-1 sections. The median number of uTAAs ranged between 2 and 4, and one uTAA was located at the start of the after-Intron-1 section overlapping with the uATG mentioned above. The median number of uTGAs ranged between 1 and 7, and uTGAs were present in both sections. A conserved in-frame uTGA stop codon at the position −9 to −7 from the sATG can be traced from primates to spotted gar with exceptions mainly in rodents and ray-finned fishes.

### 3.4. Upstream ORF Starting with Highly Conserved Upstream ATG Expanded during Vertebrate Evolution and Became Overlapping with Main ORF

Prediction of possible ORFs within the human *ABCA1* transcript showed the first ORF (entitled ORF1) on the positive strand starting at position 307 from TSS (uATG discussed above) and ending at 417. The length was calculated to be 111 nts, possibly coding for a 36 aa long protein. The length of the uORF gradually increases from 30 nts (9 aa) in coelacanth to 111 nts (36 aa) in primates (Table 1). In human, this predicted uORF overlaps with the known mORF (predicted as ORF4) which starts at position 396 (Supplemental Figure S6). Corresponding uORFs in *ABCA1* gene orthologs also overlap with known mORFs in other primates; however, with the exception of rabbit, they end before the start of mORFs in all other species evaluated. The distance between the uORF stop codon and sATG was less than 10 nts for rodents, placental mammals and platypus, less than 20 for birds, and between 20 and 30 for marsupials, anole lizard, and coelacanth. A putative bioactive peptide of 36 aa based on uORF going from 307 to 417 was also predicted by uPEPperoni program; no other short peptide was predicted within the *ABCA1* cDNA sequence.

Nucleotide contexts of the uATGs (at the start of the uORF predicted) and sATGs (at the start of the mORF annotated) were compared. The uATGs had weak nucleotide context with thymidine (T) at −3 position and adenine (A) at +4 from primates to platypus, T at −3 and cytosine (C) at +4 in birds, T at −3 and A at +4 in anole lizard, and T at −3 and T at +4 in coelacanth. The sATGs had strong context with A or G at −3 and G at +4 from primates to anole lizard, and adequate context with A at −3 and A at +4 in coelacanth (Table 2). The results of WeakAUG program are shown in Supplemental Figure S7.

### 3.5. GC Content Showed Great Variability among 5′UTRs of Extant Vertebrates with Placental Mammals Having the Highest Percentage

A high GC content of 60.8% was calculated for the whole *ABCA1* 5′UTR region in human; however, in the analysis of subsections the before-Intron-1 section showed 58.7% GC content and after-Intron-1 section 67.4%. Approximately the same percentage of GC content for the whole 5′UTR region was also calculated for the other primates, rodents, and placental mammals analyzed. In marsupials the GC content was between 53% and 58%, platypus 52.6%, reptiles and birds between 46 and 68%, coelacanth 34.8%, and ray-finned fishes between 41% and 44%. A small positive correlation between GC content and whole 5′UTR length was not significant. A more than 8% difference between the two subsections, in favor of the after-Intron-1 section, was, in addition to human, seen in opossum, coelacanth, and ray-finned fishes. On the other hand, a more than 14% difference, in favor of the before-Intron-1 section, was seen in chicken and anole lizard (Supplemental Table S5). In conclusion, GC content of whole *ABCA1* 5′UTR regions as well as subsections showed a great variability between extant vertebrate subgroups with placental mammals having the highest percentage.

### 3.6. Motif Discovery Analyses Showed Several Highly Conserved Elements Mainly among Transcriptional Regulatory Motifs and Intron Splicing Enhancers

Except for the uORFs revealed by the alignment analyses and Internal Ribosome Entry Site (IRES) in the 5′UTRs of rabbit and anole lizard, the UTR motif analysis performed by the UTRScan pattern matcher found no other conserved regulatory element.

The analysis of RNA motifs and sites calculated by RegRNA 2.0 platform was more efficient and revealed a higher number of conserved elements of different types: exon and intron splicing enhancers and silencers (ESE, ESS, ISE, and ISS, respectively), transcriptional regulatory motifs (TRM) as well as microRNA target sites. Among the most conserved elements, these were revealed: ESE—sc35 (in 9/15 species), ESS—fibronectin eda exon (7/15), ISE—ighg2 cgamma2 (7/15), ctnt (8/15) and gh-1 (10/15), TRM—TFIIA (7/15), TATA (8/15), NFAT1 (8/15), NFAT4 (8/15) and HOXA13 (8/15), and microRNA target sites—hsa-miR-4474-3p (9/15). Results of this analysis are summarized in Table 3. Detailed information about the elements found within the human *ABCA1* 5′UTR including exact sequences and their positions is provided in Supplemental Table S6.

**Figure 6:**
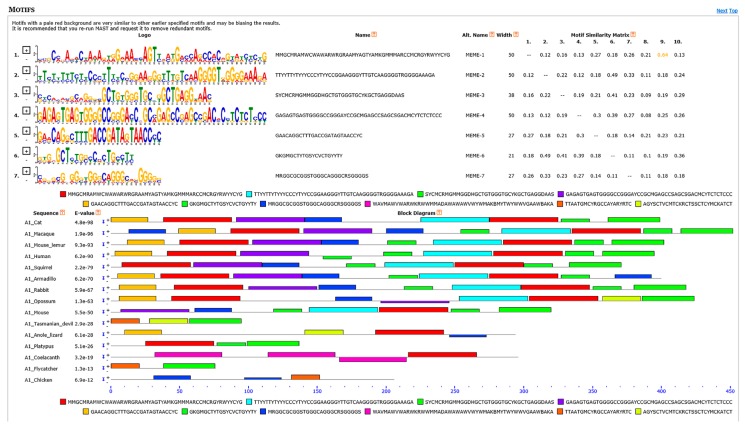
Results of DNA motif analyses performed in Motif Alignment and Search Tool (MAST), part of motif-based sequence analysis tools (MEME). 5′UTR DNA sequences of 15 vertebrate species were evaluated. Sequence logos of the seven significant motifs discovered by the program are shown in the upper part of the figure. Colors were assigned to 10 motifs found (7 significant and 3 not significant) and their distribution along the 5′UTRs is shown in the bottom part of the figure. Conservation of the motif distribution along 5′UTRs can be seen and a pattern recognized.

Based on visual observations of the multi-FASTA format files containing all the 5′UTRs downloaded from Ensembl, after base coloring, conserved subregions, which were rich for a specific base repetitions, were identified. At least from placental mammals, these base-repetition-rich subregions were distinguishable and their positions conserved. Starting from the 5′ end of the 5′UTRs we defined these subregions: G/C/A-repetition-rich subregion, G/C-repetition-rich subregion 1, T/C-repetition-rich subregion, G/A-repetition-rich subregion, and G/C-repetition-rich subregion 2 (Supplemental Figure S8). The G/A-repetition-rich subregion was localized at the end of the before-Intron-1 section (preceding Intron 1), and the G/C-repetition-rich subregion 2 spanned the whole after-Intron-1 section (preceding the start of the mORF). This view of 5′UTR segmentation was supported by the motif analyses conducted by MEME Suite tools. The MEME program discovered 7 significant DNA motifs, 21 to 50 nts long, with E-values less than 0.05. These motifs and their distribution and conservation among the 15 vertebrate species evaluated are visualized in Figure 6.

### 3.7. A Similar Number of Hairpin Loops and Their Distribution in 5′UTRs is Present throughout Extant Vertebrates

**Figure 7:**
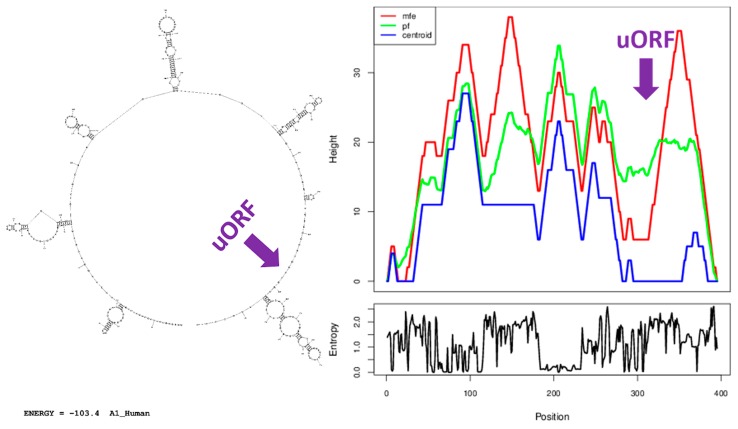
Secondary structure prediction of the human *ABCA1* 5′UTR; two web servers—RNAstructure (secondary structure diagram) and RNAfold (mountain plot) were independently employed for this analysis. A mountain plot representation of the minimum free energy prediction of secondary structure (mfe), the thermodynamic ensemble of RNA structures (pf), and the centroid structure (centroid) is presented. A mountain plot represents secondary structure in a plot of height versus position, where the height m(k) is given by the number of base pairs enclosing the base at position k, i.e., loops correspond to plateaus (hairpin loops are peaks) and helices to slopes. Additionally, the positional entropy for each position is shown (the higher the entropy, the lower the structural stability). The start position of the upstream ORF (uORF) revealed and discussed in the text is marked with an arrow.

It was predicted from the base sequence that the human 5′UTR sequence contains seven hairpin loops; six are located within the before-Intron-1 section and one within the after-Intron-1 section at the following positions (from the TSS): (+21)-(+53), (+66)-(+115), (+123)-(+154), (+156)-(+225), (+229)-(+277), (+286)-(+295), and (+312)-(+388). The hairpin loop within the after-Intron-1 section closely followed the start codon of the uORF discussed above. There were obvious differences in the size and structure of the seven hairpin loops; the hairpin loop within the after-Intron-1 section was the largest one. The optimal secondary structure with a minimum free energy of −103.4 kcal/mol was predicted (Figure 7). The minimum free energy for the optimal secondary structure of the segment of 50 nts preceding the start codon of the mORF was calculated to be −25.6 kcal/mol. A similar number and distribution of hairpin loops was seen in 5′UTRs throughout extant vertebrates sampled here (Table 4; Supplemental Figure S9).

### 3.8. Analysis of 5′UTR Variants Annotated in Ensembl Database

Analysis of human ABCA1 5′UTR variants annotated in Ensembl versus the most conserved elements defined by the study found that 24% (39 out of 161) of nts with variants were located within the study hot spots. There was no variant annotated to the start of the uORF discussed above. Results of this analysis are visualized in Supplemental Figure S10.

## 4. Discussion

In humans, *ABCA1* codes for a 2261-amino acid long protein, which is mainly localized in the plasma membrane and has the ability to translocate HDL and other substrates out of the cell. Although ABCA1 and other proteins from ABC family play key roles in many basic physiological processes, information about their 5′UTRs, which play the main role in translation efficiency management, is scarce. The current bioinformatics study reveals the precise 5′UTR characteristics for the human *ABCA1* gene and many orthologous vertebrate genes. The approach described in the text can be applied to any other gene of interest and help researchers in significant sequence finding. In the following discussion, we aim to put our observations on single gene level into the broader context of principles known from the research conducted on the whole genome level.

### 4.1. 5′UTR Length

Among *ABCA1* gene transcripts from 55 vertebrate species evaluated, longer whole 5′UTR regions, including the before-Intron-1 and after-Intron-1 sections as well as the whole Intron 1, were seen in primates and their closest relatives, with more evolutionarily distant vertebrates having all these 5′UTR parts shorter in length. In contrast, the length of the after-Intron-1-section appears to have been conserved across verterbate evolution, since it did not vary significantly among vertebrate groups studied.

The first reports comparing the average length of whole UTR regions on a whole genome level included species from very distant taxa with a limited number of vertebrates and evaluated hundreds of 5′UTRs sequenced [30,31,32]. Their attention was paid mainly to the length of 3′UTRs, which are strikingly longer and show greater variability between lineages than the average length of 5′UTRs. The increase in average length of 3′UTRs with evolutionary distance, prominent during evolution of vertebrates, was discussed and a possible link to the increase in organism complexity was proposed [30,31,32]. Based on the comparison of the average length of 5′UTRs it was concluded that the length of 5′UTRs stays relatively constant during evolution [30,31,32].

Lynch and colleagues (2003, 2005) [33,34] introduced the idea that the features of 5′UTRs, including the length, in most eukaryotes are largely dictated by random genetic drift and mutational processes that cause stochastic turnover in transcription start sites (TSSs) and premature start codons in relation to the reduced effective population sizes of eukaryotes compared to prokaryotes. For eukaryotes, lengthy 5′UTRs impose a mutational burden on their associated alleles by enhancing the rate of acquisition of a premature start codon. Natural selection would select against a long 5′UTR because of the increased rate of premature start codons and for that reason only indirectly influence the lengths of UTRs [34]. However, the idea that the variability in genome size or any of its components can be neatly explained by a single factor such as population size was questioned by several researchers at the theoretical as well as methodological levels [35,36,37]. Vinogradov and Anatskaya [38] reported that human 5′UTRs are on average 37% longer than the corresponding mouse ones and this difference remained strongly significant also after correction for 16% difference in genome sizes. The latter fact suggests according to Vinogradov and Anatskaya [38] that the difference in 5′UTR lengths cannot be just due to mutation pressure (if one assumes that the difference in genome sizes was caused by neutral drift), and should have functional significance. Compared to mouse, the longer 5′UTRs of human mRNAs as well as the 30% greater number of uAUGs, which suggest a more complexly regulated translation, are in good agreement with the higher expression rates of human translation machinery [38]. The dataset from Lynch and Conery’s work [33] was revisited with a phylogenetic perspective by Whitney and Garland [39]. In parallel, the correlation between the length of 5′UTR and organismal complexity, measured as the number of cell types, was not confirmed in the work of Chen et al. [40]; however, data from only 17 species, including 9 vertebrates, 5 invertebrates, 2 plants, and a yeast were evaluated in the study. The results of the work by Chen et al. also raise the questions of how organismal complexity can be measured and if e.g., the number of cell types is a correct proxy factor. Furthermore, Chen et al. [41] demonstrated that genomic features other than uAUGs, particularly upstream stop codons (uSTOPs) and G+C content, play an important role in the evolution of 5′UTR length. Considering that uAUGs and uSTOPs together can form uORFs, the observation of Chen et al. seems to imply that the major target of selection in 5′UTRs is likely uORFs, rather than uAUGs per se.

In a study focused on the impact of exon-exon junctions (left after intron splicing) and uORFs in 5′UTRs on gene expression Lim and colleagues [42] described the conservation of these features between human and mouse. Our conclusions about the prominent conservation of the after-Intron-1 sections are in line with these recently published results.

### 4.2. Intron 1—Length and Position

Intron 1 was annotated within the 5′UTRs in 45 species of our dataset and in 10 species there was no 5′UTR intron. Because Intron 1 was described in the representatives of the most ancient groups evaluated, spotted gar and coelacanth, the most parsimonious conclusion is that it was lost in those 10 vertebrate species independently during the evolution of *ABCA1* genes. In general, the length of Intron 1 was the longest in primates and shortest in ray-finned fishes; however, several atypically long (e.g., in coelacanth and anole lizard) or short (e.g., in chicken and cow) versions were noticed. Importantly, the position of Intron 1 has been more conserved in relation to the start codon of the main open reading frame (sATG, mORF) than to the TSS.

Studies evaluating 5′UTRs on the whole genome level have revealed that approx. 25% of metazoan 5′UTRs carry introns, with lower frequencies in plants (14%) and fungi (5%) [31,43]. Data from these studies also suggest the existence of a strong barrier against carrying more than 1 intron within 5′UTRs for all taxa. Early analyses on genomes of eukaryotic model organisms also indicated a positive correlation between genome size and intron sizes; however, because of the low level of significance, they suggested that there may be other factors involved [44,45]. Because UTR regions are under a less stringent substitutional constraint than the protein-coding sequences (CDSs), they show a higher frequency of insertions and deletions and greater length heterogeneity [46,47]. As a consequence, introns in UTRs may experience less stabilizing selection for some traits than introns in CDSs; the more dynamic nature of UTRs may thus promote intron loss and result in lower intron abundance in comparison with CDSs [43]. Hong and colleagues [43] suggested a mechanism for the occurrence of selection differences on intron size in 5′UTRs versus CDSs that may occur over very short distances, driven by the potentially deleterious effects of uAUGs within 5′UTR exons. They proposed the existence of: (1) selection against intron contraction, due to the potential introduction of uAUGs residing in 5′UTR introns at nearly neutral proportions; and (2) selection for intron expansion, due to the beneficial effects of both removing uAUGs from 5′UTR exons and preventing the appearance of new uAUGs by reducing the total 5′UTR exon length.

Another view was given by Vinogradov [48], who hypothesized that longer introns (as well as intergenic regions) preferentially occur in tissue-specific genes because they allow chromatin-mediated gene suppression and complex regulation. The data reported by Pozzoli et al. [49] supported and extended this view. In particular, their unifying model proposed that regulatory needs, accounted for by multi-species conserved sequences (MCSs), shape intron size and tend to be stronger in genes that are not highly expressed. They also showed that, when MCS content was fixed, no variation of size with expression level was observed for introns (and intergenic spacers) in genes expressed at a medium-to-low level. They therefore proposed that the fixation of functional conserved elements is the adaptive event underlying size increase [49].

We have shown that the increase in length of the whole region spanning *ABCA1* 5′UTRs studied can be attributed mainly to the striking increase in Intron 1 length. Similar observations on the gene level were published by LaConte and Mukherjee [50] in their work describing the major constraints that have shaped the evolution of *CASK* (Ca^2+^/calmodulin-activated serine kinase) genes. They showed that despite the tremendous increase in the size of the *CASK* gene over the course of animal evolution, the changes in the number of introns have been minimal, and most of the gene size increase can be attributed to an increase in the size of the introns.

### 4.3. Upstream ORF—Position, Function, and Evolution

Within the *ABCA1* 5′UTRs we defined a 15 nts long sequence consisted of the most conserved nucleotides based on alignment analyses. This sequence was almost always located at the start of the after-Intron-1 section. Only one uATG was found within the whole 5′UTRs evaluated with only a few exceptions having none or more than one, mainly within ray-finned fishes. This out-of-frame uATG was assigned to the most conserved 15 nts long sequence. Several bioinformatics tools predicted an uORF which starts from this uATG. The length of the uORF and protein possibly coded by this regulative feature varies from 30 nts, i.e., 6 aa, in coelacanth through 51 nts, i.e., 16 aa, in anole lizard; and 78 nts, i.e., 25 aa, in platypus to 111 nts, i.e., 36 aa, in primates where the uORF started to overlap with mORF. A highly conserved in-frame uTGA stop codon at the position −9 to −7 from the sATG was described within the 5′UTRs from primates to ray-finned fishes. Moreover, we showed striking differences in the distribution of upstream non-ATG start and stop codons within the 5′UTR sections studied.

Generally, uATGs decrease mRNA translation efficiency and may be considered to be negative translational regulatory factors [51]. Among five commonly studied vertebrate species including human, a median of 36% of 5′UTRs contained at least one uATG [52]. A higher percentage of uATG incidence (44%) was calculated several years later by Iacono et al. [53] and an even higher percentage (49%) by Calvo et al. [54] in human and rodent 5′UTR transcripts. Although uATGs were shown to be relatively common, the frequencies of ATG trinucleotides in 5′UTRs were significantly lower than the frequencies expected by chance, suggesting that ATGs are specifically selected against in 5′UTRs and can bear an important functional relevance [52,53]. Based on the comparison of human, mouse, and rat as well as different species of the yeast genus *Saccharomyces* orthologous genes, Churbanov and co-workers [55] concluded that ATG triplets in 5′UTRs are subject to the pressure of purifying selection in two opposite directions: the uATGs that have no specific function tend to be deleterious and get eliminated by natural selection, whereas those uATGs that do serve a function are conserved. Most probably, the principal role of the conserved uATGs is attenuation of translation at the initiation stage by diverting scanning ribosomes from the authentic sAUGs. This process is often additionally regulated by alternative splicing in the mammalian 5′UTRs [55]. Consistent with this hypothesis, they found that ORFs starting from conserved uATGs are significantly shorter than those starting from non-conserved uATGs. However, because they compared only sequences from relatively closely related species, they could not recognize the pattern of increase in uORF length among vertebrate species as we described for the first time in this study for the *ABCA1* orthologous genes. Matsui et al. [56] introduced the idea that uORFs are sequence elements that downregulate RNA transcripts via RNA decay mechanisms which maintain the balance between the synthesis and degradation of RNA transcripts.

The application of alternative promoters and alternative splicing are two well-known mechanisms contributing to protein isoform complexity. Another less explored molecular mechanism allowing several protein variants to be produced from a single mRNA is alternative translation. Based on experimental work, it has been assumed that ribosomes can recognize alternative translation initiation sites (TISs) by leaky scanning and additional protein isoforms can differ in functional properties [57,58]. The initiation/scanthrough ratio depends on the ATG nucleotide context. When the preferred first ATG resides in a weak context, lacking both purine (R) in position −3 and guanine (G) in +4, some ribosomes initiate at that point but most continue scanning and initiate farther downstream [51]. This situation may be true for the uORF defined in this study, which starts from the first ATG with a weak context, allowing mORF, which starts from the second ATG with a strong context, to be read in most cases. Similarly, reinitiation of translation after a short uORF can be employed as a second mechanism for selection of alternative TISs and synthesis of alternative protein isoforms [57,58]. In addition, reinitiation occurs more efficiently at longer distances between the stop codon of the uORF and the next start ATG [59,60]. In our case, the uORF is small enough to allow reinitiation; however, the distance between the uORF stop codon and sATG is quite short and in primates the uORF even overlaps with the mORF. It can be speculated that reinitiation in *ABCA1* genes is possible; however, a different isoform from the main one is produced starting from an ATG further downstream. Based on bioinformatics analyses, Kochetov et al. [61] assumed that some 5′UTR-located uORFs can function to deliver ribosomes to alternative TISs, and they should be taken into consideration in the prediction of human mRNA coding potential. Moreover, it has been shown experimentally that gene expression can be determined by a combination of leaky scanning and reinitiation events and the system is sensitive to changing global translational conditions, e.g., the expression of the yeast transcription factor GCN4 under nutritional stress conditions [62]. uORFs overlapping with mORFs (VuORFs) were reported to participate in the regulation of condition-specific protein expression by Hsu and Chen [63].

As uORFs often reduce the rate of translation of the main isoform, the possibility of a SNP creating or removing an uORF would be expected to have serious consequences on gene expression level. Verified examples of such disease-causing SNPs in humans, congenital as well as acquired, were reviewed in, e.g., Barbosa et al. [64] and Somers et al. [65]. We plan to focus on this issue in ABC genes during our ongoing next-generation sequencing experiments. Furthermore, much emerging evidence about functional short peptides (sPEPs) encoded by short ORFs (sORFs), which can be located at any gene section, has broadened our understanding of transcriptomic and proteomic complexity [66,67]. A putative bioactive peptide of 36 aa based on the above-described uORF in *ABCA1* gene was also predicted by the uPEPperoni program.

### 4.4. GC Content and Motifs

The GC content of the whole 5′UTRs as well as subsections showed great variability between extant vertebrate subgroups and in some cases even within closer species. Placental mammals showed the highest percentage of GC content. Motif discovery analyses revealed several highly conserved elements among transcriptional regulatory motifs (TFIIA, TATA, NFAT1, NFAT4, and HOXA13), exon and intron splicing enhancers (sc35, ighg2 cgamma2, ctnt, and gh-1), exon splicing silencers (fibronectin eda exon) as well as microRNA target sites (hsa-miR-4474-3p). The existence of specific base-repetition-rich subregions which were conserved and created a characteristic pattern within the 5′UTRs was suspected from visual screening of the sequences and MEME program results.

The mammalian genome is characterized by its high spatial heterogeneity in base composition. The average GC content of a 100-kb fragment of the human genome can be as low as 35% or as high as 60%, a range that is twice as wide as that typically observed in teleost fishes [68,69]. Several published studies have explained the cross-species variability in GC content at the genome level, e.g., Duret et al. [70], Gu and Li [71], and Romiguier et al. [69]. Reuter et al. [37] did not see a positive relationship between GC content and 5′UTR length in genomic data from yeast, fruit fly, and human. We also did not see this correlation on our data; however, the data set tested was quite small.

Transcription of RNA polymerase II-dependent genes is triggered by the regulated assembly of the preinitiation complex. Preinitiation complex formation commences with the binding of transcription factor IID (TFIID), which contains the TATA-box binding protein (TBP), to the core promoter. Binding of TFIID to the core promoter is followed by the recruitment of further general transcription factors, including transcription factor IIA (TFIIA), and RNA polymerase II [72]. The finding of high conservation of TF and TATA elements within *ABCA1* 5′UTRs is consistent with their importance for transcription initiation. The NFAT (nuclear factor of activated T cells) family of transcription factors consists of four Ca^2+^-regulated members (NFAT1–NFAT4) and one protein—NFAT5, which is activated in response to osmotic stress. In addition to their well-documented role in T lymphocytes, where they control gene expression during cell activation and differentiation, NFAT proteins are also expressed in a wide range of cells and tissue types and regulate genes involved in cell cycle, apoptosis, angiogenesis, and metastasis. The NFAT proteins share a highly conserved DNA-binding domain, which allows all NFAT members to bind to the same DNA sequence in enhancers or promoter regions [73]. The homeobox (HOX) genes are an evolutionarily highly conserved gene family of homeodomain-containing transcription factors, which play critical roles in regulating embryonic development. HOXA13, which is a member of the Abd-B subfamily of Hox genes, is crucial for the autopod development of the limb. Increasing evidence now indicates that dysregulated expression of HOX genes, including HOXA13, in a variety of cancers is closely related to tumor development and progression [74]. The high conservation of NFAT and HOXA elements can be probably attributed to the ABCA1 transport function and suggests a connection between ABCA1 protein and cancer [75]. We can speculate that those base-repetition-rich subregions observed in this study play more general roles in translation initiation and/or recognition of introns and initiate further study in this issue.

### 4.5. Secondary Structure

In contrast to GC content, the number and distribution of hairpin loops was found to be relatively conserved across vertebrates. Notably, the largest hairpin loop structure followed closely the upstream start codon of the uORF predicted in the study.

It has been proposed that stable secondary structures can influence TIS recognition. A hairpin loop structure located downstream of the start codon could delay 40S ribosomal subunit movement into the proper position and facilitate the recognition of TIS in a weak context [76,77]. Understanding of this issue of secondary structure involvement is still limited and experiments testing the influence of different hairpin loops located closely downstream on uORF translation need to be conducted.

Secondary structure of mRNA affects translation in more than one way [78]. Significant correlations between folding free energies of 5′UTRs and various transcript features were found measured in genome-wide studies of yeast [79]. In particular, mRNAs with weakly folded 5′UTRs have higher translation rates, higher abundances of the corresponding proteins, longer half-lives and higher numbers of transcripts. According to our predictions, human *ABCA1* 5′UTR is strongly folded, because it has a secondary structure with a low value of the minimum free energy. Similar situations can be found also in other vertebrates. Transcripts with 5′UTRs that hamper their translation often encode for proteins that need to be strongly and finely regulated.

In conclusion, we have defined highly conserved sequences within the 5′UTRs of vertebrate *ABCA1* orthologous genes, including human. Elements having the strongest influence on transcription or translation of this gene—exon and intron enhancers and silencers (sc35, ighg2 cgamma2, ctnt, gh-1 and fibronectin eda exon), transcription factors (TFIIA, TATA, NFAT1, NFAT4, and HOXA13), microRNAs (hsa-miR-4474-3p), and uORF and secondary structure features—were located to these sequences. Single nucleotide polymorphisms (SNPs) disrupting these important elements can probably have an impact on *ABCA1* gene expression. We calculated that 24% of the nts with variants annotated in Ensembl database were located within these study hot spots. Furthermore, the uORF characterized in the study possibly codes for an unknown bioactive peptide (sPEP). We described a work-flow which can be suitably applied to any other gene of interest based on freely available bioinformatics tools (Table 5). We hope that it can help researchers dealing with next-generation sequencing results in case of research as well as clinical purposes. Moreover, we showed several features of 5′UTRs which are interesting from a phylogenetic point of view and can stimulate further evolutionary oriented research.

## Figures and Tables

**Table 1:** Comparison of the conserved uORF predicted in ABCA1 orthologous genes.

Species	uORF					mORF
	Start	Stop	Length		Protein Sequence	Start
			nts	aa		
Human	307	417	111	36	MTSHGVPAVSSGRCLPGLPSHTLGVLAEGTWLVGLS	396
Macaque	364	474	111	36	MTSHGIPAVSSGRCLPGLLSHTLWVLAEGTWLVGLS	453
Mouse lemur	314	424	111	36	MTSHSIPAVSSGHCLPGLLSHTLWVPAEVTWLVGPS	403
Rabbit	327	440	114	37	MTSHSGFATSSGRCLQGRATSRLPWVPAEVTWPAGLS	419
Mouse	224	319	96	31	MTSHRVTALCSGCSLQGSRAADAGRCGCRLW	321
Squirrel	279	365	87	28	MTSHSVCCELRPVPPGLLSHTQVALGAG	372
Cat	304	393	90	29	MTSHSVPAVSCCCCLQKLLSHTQVAAAAG	400
Armadillo	304	399	96	31	MTSHSVPAVSSGHCPHGLPTSHTQVAWARLR	401
Tasmanian devil	4	66	63	20	MTSHSVPAQRYLCSLHYLPG	96
Opossum	333	395	63	20	MTSHGVLAQCCLCSLHYLLD	425
Platypus	54	131	78	25	MTSHSVPAVCCCHCPCHTRGAVPAC	138
Chicken	135	200	66	21	MPSHNVLVVYCCCCTKGRRHC	207
Flycatcher	4	60	57	18	MPGHNICTVLLLLHKESF	77
Anole lizard	221	271	51	16	MTSHSSSAVCCFHPRC	295
Coelacanth	245	274	30	9	MSDNNIPAA	297

Note: Start/Stop positions from transcription start site.

**Table 2:** Comparison of uORF and mORF start nucleotide contexts in ABCA1 orthologous genes.

Species	uORF nt Context	mORF nt Context
Human	AAACAGTTAATGACCAGCCAC	TGAGGGAACATGGCTTGTTGG
Macaque	AAACAGTTAATGACCAGCCAC	TGAGGGAACATGGCTTGTTGG
Mouse lemur	AAGCAGTTAATGACCAGCCAC	TGAGGTGACATGGCTTGTTGG
Rabbit	AAGCAGTTAATGACCAGCCAC	TGAGGTAACATGGCCTGCTGG
Mouse	AAACAGTTAATGACCAGCCAC	TGTGGTGACATGGCTTGTTGG
Squirrel	AAACAGTTAATGACCAGCCAC	TGAGGTAACATGGCTTATTGG
Cat	AAACAGTTAATGACCAGCCAC	TGAGGAAACATGGCTTACTGG
Armadillo	AAACAGTTAATGACCAGCCAC	TGAGGTAACATGGCTTGCTGG
Tasmanian devil	TTAATGACCAGCCAC	TGAGGAAAGATGGCTTTTTGG
Opossum	AAGCAGTTAATGACCAGCCAC	TGAGGAGAGATGGCCTTTTGG
Platypus	TTCCAGTTAATGACCAGCCAC	TGAGGAAAGATGGCTTTTTGG
Chicken	CCGGAGTTAATGCCCAGCCAT	TGAAGAACGATGGCATTTTGG
Flycatcher	TTAATGCCTGGCCAC	TGAAGGAAGATGGCTTTCTGG
Anole lizard	GAGGAGTTGATGACCAGCCAC	AGAAGGAAGATGGCCTTCTGG
Coelacanth	AAAAAGTTAATGTCCGACAAC	TGGGAAAAGATGACTTTCTGG

Abbreviations: nt, nucleotide; ATG codons are highlighted in red font.

**Table 3:** Analysis of conserved RNA motifs and sites in ABCA1 5′UTRs of 15 vertebrate species.

		Species															
Motif Type	Motif Name	Hu.	Ma.	Mo. le.	Sq.	Mo.	Ra.	Cat	Ar.	Ta. de.	Op.	Pl.	Chi.	Fl.	An. li.	Co.	Nr. of Hits
ESE	beta-globin ex. 2											•			•		2
	ct/cgrp	•	•					•									3
	**sc35**	•	•	•	•	•	•	•	•				•				9
ESS	**fibronectin eda ex.**	•	•		•		•	•			•				•		7
ISE	cftr, in. 9					•	•								•		3
	**ighg2 cgamma2 in. 1**	•	•	•	•			•	•				•				7
	**ctnt, ex. 5**	•	•	•	•	•		•	•	•							8
	**gh-1 in. 3**	•	•	•	•	•	•	•	•		•		•				10
rho-ind. ter.	Rho-ind. ter.	•	•	•				•									4
TRM	Sp1	•	•														2
	Msx-1	•	•														2
	Freac		•													•	2
	c-Ets-1_p54						•	•									2
	SMAD								•							•	2
	GATA-5									•	•						2
	ZNF333										•				•		2
	ELF1		•											•	•		3
	SPI1		•											•	•		3
	MZF1			•		•										•	3
	MYB			•					•		•						3
	AP-2			•				•	•								3
	BEN						•	•	•								3
	Kid3	•	•						•		•						4
	PEA3	•	•				•	•									4
	E2F-3	•	•	•			•										4
	GABP		•	•			•	•									4
	ZF5			•		•		•							•		4
	SOX10												•	•	•	•	4
	Elk-1	•	•		•		•	•									5
	ER81	•	•		•		•	•									5
	ETV7	•	•		•		•	•									5
	LXR_direct			•			•	•	•		•						5
	**TFIIA**	•	•	•	•			•	•		•						7
	**TATA**	•	•	•	•			•	•		•					•	8
	**NFAT1**	•	•		•	•	•	•	•							•	8
	**NF-AT4**	•	•		•	•	•	•	•							•	8
	**HOXA13**	•	•	•	•			•	•		•					•	8
UTR motifs	Musashi bin. El. (MBE)	•	•	•			•	•	•								6
microRNA target sites	hsa-miR-5581-5p	•	•														2
	hsa-miR-3194-3p		•										•				2
	hsa-miR-4435	•	•	•													3
	**hsa-miR-4474-3p**			•	•	•	•	•	•	•		•			•		9

Abbreviations: bin, binding; el., element; ESE, exon splicing enhancer; ESS, exon splicing silencer; ex., exon; in., intron; ind., independent; ISE, intron splicing enhancer; Nr., Number; ter., terminator; TRM, transcriptional regulatory motif; UTR, untranslated region; Species Abbreviations: Hu., human; Ma., macaque; Mo. le., mouse lemur; Sq., squirrel; Mo., mouse; Ra., rabbit; Cat, cat; Ar., armadillo; Ta. de., Tasmanian devil; Op., opossum; Pl., platypus; Chi., chicken; Fl., flycatcher; An. li., anole lizard; Co., coelacanth; •—motif was found within the relevant 5′UTR in one or more copies; the most important motifs are highlighted in bold;

**Table 4:** Analysis of ABCA1 5′UTR secondary structures in RNAstructure and RNAfold programs.

Species	No. of Hairpin Loops			
	RNAstructure		RNAfold	
	Before In. 1 Section	After In. 1 Section	Before In. 1 Section	After In. 1 Section
Human	6	1	6	1
Mouse lemur	4	1	5	2
Rabbit	7	1	7	1
Mouse	5	1	4	2
Armadillo	7	1	5	1
Opossum	7	1	5	1
Platypus	2		2	
Flycatcher	2		2	
Anole lizard	7	1	6	1
Coelacanth	6	1	4	1

Abbreviations: In., intron; No., number.

**Table 5:** Summary of software and database resources.

**DNA and RNA Sequences of Multiple Species**	
**Ensembl, UCSC**	genome browsers for the retrieval of genomic information
**Taxonomy Browser (NCBI)**	builds trees based on the classification in the NCBI taxonomy database
**iTOL**	phylogenetic tree development and visualisation
**EMBOSS Transeq**	translates nucleic acid sequences to their corresponding peptide sequences
**Transcription and Translation Tool (ExPASy)**	transcription, translation, reverse transcription
**Alignment analyses**	
**Jalview**	multiple sequence alignment editing, visualisation and analysis
**EMBL-EBI, UCSC, NCBI**	bioinformatics resources including alignment programs
**Upstream start and stop codons and ORF prediction**	
**ORF Finder**	searches for open reading frames in DNA sequences
**NetStart**	produces neural network predictions of translation start in nucleotide sequences
**uPEPperoni**	location and identification of upstream open reading frames that have the potential to encode bioactive peptides
**WeakAUG**	predict the initiation site of mRNA sequences that lack the preferred nucleotides
**GC content calculation and motif discovery**	
**GC Content Calculator**	GC content, GC Content Distribution Plots
**UTRscan**	finds motifs that characterize 3′UTR and 5′UTR sequences
**RegRNA**	identifies functional RNA motifs and sites in RNA sequences
**MEME**	discovers novel motifs in nucleotide or protein sequences
**Secondary structure prediction**	
**RNAstructure, RNAfold**	predict RNA secondary structures
**Statistics**	
**PAST**	scientific data analysis, statistics

## Supplementary Materials

The following are available online at https://www.mdpi.com/2073-4409/8/6/623/s1. Supplemental Table S1: Multi-FASTA format file containing *ABCA1* 5′UTR sequences of 55 species (59 transcripts without intron sequences) downloaded from the Ensembl database and evaluated within comparative analyses. Supplemental Table S2: Summary of all species and names and IDs of transcripts downloaded from the Ensembl database, lengths of 5′UTR sections evaluated—whole 5′UTR, 5′UTR section located before-Intron-1, section located after-Intron-1, whole Intron 1 lengths, and ABCA1 protein lengths in amino acids. Supplemental Table S3: Summary of multiple-sequence (59 transcripts) alignment analyses performed with the help of 9 alignment programs. Highly conserved nucleotides are those with >80% identity and >84% occupancy scores. Supplemental Table S4: Upstream ATG and non-ATG start as well as stop codon comparison—number and position. Supplemental Table S5: GC content versus length comparison in 5′UTRs of orthologous *ABCA1* genes. Supplemental Table S6: Analysis of conserved RNA motifs and sites in human ABCA1 5′UTR—detailed information. Supplemental Figure S1: The *ABCA1* gene tree created by the Gene Tree tool—Ensembl database. Supplemental Figure S2: Results of the ABCA1 5′UTR multi-sequence alignment analysis in the ClustalO program performed on 59 transcripts downloaded from the Ensembl database. Lines represent transcripts, which were aligned according to the ClustalO algorithm; the conservation of nucleotides among vertebrate species is highlighted with the most conserved ones having the darkest color. A) Alignment of the whole 5′UTRs. 5′UTR segments, which contain conserved nucleotides can be distinguished. B) Detail of the 5′UTR segment containing the most conserved sequences; the position of the spliced Intron 1 is marked by a red arrow. Supplemental Figure S3: Results of the conservation track focused on the 5′UTR region of the human *ABCA1* gene performed in UCSC Genome Browser. Three highly conserved regions (HCR1-HCR3) can be distinguished. HCR3 is overlapping with the most conserved sequence displayed on Figure 5 in the main text. Supplemental Figure S4: The analysis of uCTG distribution within 5′UTRs of *ABCA1* genes from 55 species, 5′UTR sequences were downloaded from the Ensembl database. Supplemental Figure S5: The analysis of uTTG distribution within 5′UTRs of *ABCA1* genes from 55 species, 5′UTR sequences were downloaded from the Ensembl database. Supplemental Figure S6: ORF prediction on the 5′ end of human *ABCA1* transcript calculated by ORFfinder. Upstream ORF discussed within the main text was predicted as ORF1 and known main ORF as ORF4. Supplemental Figure S7: Results obtained from the WeakAUG server. Supplemental Figure S8: Analysis of specific base-repetition-rich subregions in 5′UTRs of orthologous *ABCA1* genes, 5′UTR sequences of 55 species were downloaded from the Ensembl database. Supplemental Figure S9: Secondary structure comparison of *ABCA1* 5′UTRs in 10 vertebrate species. Two web servers—RNAstructure (secondary structure diagrams) and RNAfold (mountain plots) were employed independently. Results of the following species are disclosed: A) human, B) mouse lemur, C) rabbit, D) mouse, E) armadillo, F) opossum, G) platypus, H) flycatcher, I) anole lizard, and J) coelacanth. Start position of the uORF discussed in the main text is marked with an arrow. Supplemental Figure S10: Analysis of the human *ABCA1* 5′UTR variants from Ensembl versus hot spots defined by the study.

## Abbreviations

5′UTR	5′ untranslated region
ESE	exon splicing enhancer
ESS	exon splicing silencer
HDL	high density lipoprotein
ISE	intron splicing enhancer
mORF	main open reading frame
nts	nucleotides
SNP	single nucleotide polymorphism
sPEP	small peptide
TIS	translation initiation site
TSS	transcription start site
uATG	upstream ATG codon
uORF	upstream open reading frame
