# Supplementary material for: Functionally Significant Features in the 5′ Untranslated Region of the ABCA1 Gene and Their Comparison in Vertebrates
Source: Cells. 2019 Jun 21;8(6):623. doi: 10.3390/cells8060623 (PMC6627321; doi:10.3390/cells8060623)
Supplement: Supplementary file 1 [file cells-08-00623-s001.pdf]

Supplementary Information 1 – Supplementary Tables

Supplemental Table S1

>A1_Chimpanzee

GGGGGCGGGGAGGAGGGAGAGCACAGGCTTTGACCGATAGTAACCTCTGCGCTCGGTGCA

GCCGAATCTATAAAAGGAACTAGTCTCGGCAAAAACCCCGTAATTGCGAGCGAGAGTGAG

TGGGGCCGGGACCCGCAGAGCCGAGCCGACCCTTCTCTCCCGGGCTGCGGCAGGGCAGGG

CGGGGAGCTCCGCGCACCAACAGAGCCGGTTCTCAGGACGCTTTGCTCCTTGTTTTTTCC

CCGGTTCTGTTTTCTCCCCTTCTCCGGAAGCCTTGTCAAGGGGTAGGAGAAAGAGACGCA

AACACAAAAGTGGAAAACAGTTAATGACCAGCCACGGCGTCCCTGCTGTGAGCTCTGGCC

GCTGCCTTCCAGGGCTCCCAAGCCACACGCTGTGGGTGCTGGCTGAGGGAAC

>A1_Bonobo

GGAGGAGGGAGAGCACAGGCTTTGACCGATAGTAACCTCTGCGCTCGGTGCAGCCGAATC

TATAAAAGGAACTAGTCTCGGCAAAAACCCCGTAATTGCGAGCGAGAGTGAGTGGGGCCG

GGACCCGCAGAGCCGAGCCGACCCTTCTCTCCCGGGCTGCGGCAGGGCAGGGCGGGGAGC

TCCGCGCACCAACAGAGCCGGTTCTCAGGACGCTTTGCTCCTTGTTTTTTCCCGGGTTCT

GTTTTCTCCCCTTCTCCGGAAGGCTTGTCAAGGGGTAGGAGAAAGAGACGCAAACACAAA

AGTGGAAAACAGTTAATGACCAGCCACGGCGTCCCTGCTGTGAGCTCTGGCCGCTGCCTT

CCAGGGCTCCCAAGCCACACGCTGTGGGTGCTGGCTGAGGGAAC

>A1_Human

AGAGCACAGGCTTTGACCGATAGTAACCTCTGCGCTCGGTGCAGCCGAATCTATAAAAGG

AACTAGTCCCGGCAAAAACCCCGTAATTGCGAGCGAGAGTGAGTGGGGCCGGGACCCGCA

GAGCCGAGCCGACCCTTCTCTCCCGGGCTGCGGCAGGGCAGGGCGGGGAGCTCCGCGCAC

CAACAGAGCCGGTTCTCAGGGCGCTTTGCTCCTTGTTTTTTCCCCGGTTCTGTTTTCTCC

CCTTCTCCGGAAGGCTTGTCAAGGGGTAGGAGAAAGAGACGCAAACACAAAAGTGGAAAA

CAGTTAATGACCAGCCACGGCGTCCCTGCTGTGAGCTCTGGCCGCTGCCTTCCAGGGCTC

CCGAGCCACACGCTGGGGGTGCTGGCTGAGGGAAC

>A1_Gorilla

GGAGGAGGGAGAGCACAGGCTTTGACCGATAGTAACCTCTGCGCTCGGTGCAGCCGAATC

TATAAAAGGAACTAGTCTCGGCAAAAACCCCGTAATTGCGAGCGAGATTGAGTGGGGCCG

GGACCCGCAGAGCCGAGCCGACCCTTCTCTCCCGGGCTGCGGCAGGGCAGGGCGGGGAGC

TCCGGGCACCAACAGGGCCGGTTCTCAGGGCGCTTTGCTCCTTGTTTTTTCCCCCGTTCT

ATTTTCTCCCCTTCTCCGGAAGGCTTGTCAAGGGGTAGGAGAAAGAGACGCAAACACAAA

AGTGGAAAACAGTTAATGACCAGCCACGGCGTCCCTGCTGTGAGCTCTGGCCGCTGCCTT

CCAGGGCTCCCGAGCCACACGCTGTGGGTGCTGGCTGAGGGAAC

>A1_Golden_s_n_monkey

GGAGGAAGGCGAGAACAAGCTTTGACCGATAGTAACCTCTGCGCTCCGGGCAGCCGAATC

TATAAAAGGAACTAGTCTCGGCAAAAACCCCATAATTGCGAGCGAGAGTGAGTGGGGCCG

GGACCCGCAGAGCCGAGCCGACCCTTCTCTCCCGGGCTGCGGCAGGGCAGGGCAGAGCAG

GGCGGGGGGCTCCGCGCACCAACAGGACCAGTTCTCAGGGCGCTTTGCTCCTTGTTTTTT

CCCCTCGTTCTGTTTTCCCCCCTTCTCCGGAAGGCTTGTCAAGGGGTAGGAGAAAGAGAC

GCAAACACAAAAGTGGAAAACAGTTAATGACCAGCCACGGCGTCCCTGCTGTGAGCTCTG

GCCGCTGCCTTCCAGGGCTCCCGAGCCACACACTGTGGGTGCTGGCTGAGGGAAC

>A1_Black_s_n_monkey

GACTGAACTACATAAACAGAGGCCGGGAAGGGGGCGGGGAGGAAGGCGAGAACAAGCTTT

GACCGATAGTAACCTCTGCGCTCCGGGCAGCCGAATCTATAAAAGGAACTAGTCTCGGCA

AAAACCCCATAATTGCGAGCGAGAGTGAGTGGGGCCGGGACCCGCAGAGCCGAGCCGACC

CTTCTCTCCCGGGCTGCGGCAGGGCAGGGCAGAGCAGGGCGGGGGGCTCCGCGCACCAAC

AGGACCAGTTCTCAGGGCGCTTTGCTCCTTGTTTTTTCCCCTCGTTCTGTTTTCCCCCCT

TCTCCGGAAGGCTTGTCAAGGGGTAGGAGAAAGAGACGCAAACACAAAAGTGGAAAACAG

TTAATGACCAGCCACGGCGTCCCTGCTGTGAGCTCTGGCCGCTGCCTTCCAGGGCTCCCG

AGCCACACACTGTGGGTGCTGGCTGAGGGAAC

>A1_Angola_colobus

GGAGGAAGGCGAGAACAGGCTTTGACCGATAGTAACCTCTGCGCTCCGGGCAGCCGAATC

TATAAAAGGAACTAGTCTAGGCAAAAACCCTGTAATTGCGAGCGAGAGTGAGTGGGGCCG

GGACCCGCAGAGCCGAGCCGACCCTTCTCTCCTGGGCTGCGGCAGGGCAGGGCAGAGCAG

GGCGGGGGGCTCTGCGCACCAACAGGACCAGTTCTCAGGGCGCTTTGCTCCTTGTTTTTT

CCCCTCGTTCTGTTTTCTCCCCTTCTCCGGAAGGCTTGTCAAGGGGTAGGAGAAAGAGAC

GCAAACACAAAAGTGGAAAACAGTTAATGACCAGCCACGGCGTCCCTGCTGTGAGCTCTG

GCCGCTGCCTTCCAGGGCTCCCGAGCCACACGCTGTGGGTGCTGGCTGAGGGAAC

>A1_Crab_e_macaque

GGGGGATTCAGTATGGAGCTCGCTCTCCCCCAATCCCTCCCTCCGGCTGAGGAAACTAAC

AAAGGGAAAAAAGATTGCGGAAAGCACGATTTAGGGGAAGCAAATTCCACTGGTGCCCTT

GGCTGCCGGGAACATGGACTAGAGAGTCTGCGGCGCAGCCCCGAGCCCAGCGCTTCCCGC

GGGTCTTAGGCCGGCGGGCCCCGGCGGGGAGCGGGGGCGCAGACCGCGGACCCTGAGACA

CCTGATACACCCTCCACCCCCACCCCACCCACCTCCTCCCAACTCCCTAGCAGCTTCGCG

GGCGGCTGAACGTCGCCCGTTTAAGAGGCGGGCCGCGGCTCCACGTGCTTTCTGCTGAGT

GACTGAACTACATAAACAGAGGCCGGGAAGGGGGCGGGGAGGAAGGCTAGAACAGGCTTT

GACCGATAGTAACCTCTGCGCTCCGGGCAGCCGAATCTATAAAAGGAACTAGTCTCGGCA

AAAACCCCGTAATTGCGAGCGAGAGTGAGTGGGGCCGGGACCAGCAGAGCCGAGCCGACC

CTTCTCTCCCGGGTTGCGGCAGGGCAGAGCAGGGCGGGGGGCTCTGCGCACCAACAGGAC

TAGTTCTCAGGGCGCTTTGCTCCTTGTTTTTTCCCCTCGTTCTGTTTTCTCCCCTTCTCC

GGAAGGCTTGTCAAGGGGTAGGAGAAAGAGACGCAAACACAAAAGTGGAAAACAGTTAAT

GACCAGCCACGGCATCCCTGCTGTGAGCTCTGGCCGCTGCCTTCCAGGGCTCCTGAGCCA

CACGCTGTGGGTGCTGGCTGAGGGAAC

>A1_Pig_t_macaque

CAGCTTCGCGGGCGGCTGAACGTCGCCCGTTTAAGAGGCGGGCCGCGGCTCCACGTGCTT

TCTGCTGAGTGACTGAACTACATAAACAGAGGCCGGGAAGGGGGCGGGGAGGAAGGCGAG

AACAGGCTTTGACCGATAGTAACCTCTGCGCTCCGGGCAGCCGAATCTATAAAAGGAACT

AGTCTCGGCAAAAACCCCGTAATTGTGAGCGAGAGTGAGTGGGGCCGGGACCCGCAGAGC

CGAGCCGACCCTTCTCTCCCGGGTTGCGGCAGGGCAGAGCAGGGCGGGGGGCTCTGCGCA

CCAACAGGACTAGTTCTCAGGGCGCTTTGCTCCTTGTTTTTTCCCCTCGTTCTGTTTTCT

CCCCTTCTCCGGAAGGCTTGTCAAGGGGTAGGAGAAAGAGACGCAAACACAAAAGTGGAA

AACAGTTAATGACCAGCCACGGCATCCCTGCTGTGAGCTCTGGCCGCTGCCTTCCAGGGC

TCCTGAGCCACACGCTGTGGGTGCTGGCTGAGGGAAC

>A1_Macaque

GACTGAACTACATAAACAGAGGCCGGGAAGGGGGCGGGGAGGAAGGCGAGAACAGGCTTT

GACCGATAGTAACCTCTGCGCTCCGGGCAGCCGAATCTATAAAAGGAACTAGTCTCGGCA

AAAACCCCGTAATTGCGAGCGAGAGTGAGTGGGGCCGGGACCAGCAGAGCCGAGCCGACC

CTTCTCTCCCGGGTTGCGGCAGGGCAGGGCAGAGCAGGGCGGGGGGCTCTGCGCACCAAC

AGGACTAGTTCTCAGGGCGCTTTGCTCCTTGTTTTTTCCCCTCGTTCTGTTTTCTCCCCT

TCTCCGGAAGGCTTGTCAAGGGGTAGGAGAAAGAGACGCAAACACAAAAGTGGAAAACAG

TTAATGACCAGCCACGGCATCCCTGCTGTGAGCTCTGGCCGCTGCCTTCCAGGGCTCCTG

AGCCACACGCTGTGGGTGCTGGCTGAGGGAAC

>A1_Drill

GAGGAAGGCGAGAACAGGCTTTGACCGATAGTAACCTCTGCGCTCCGGGCAGCCGAATCT

ATAAAAGGAACTAGTCTCGGCAAAAACCCCGTAATTGCGAGCGACAGTGAGTGGGGCCGG

GACCCGCAGAGCCGAGCGGACCCTTCTCTCCCGGGTTGCGGCAGGGCAGAGCAGGGCGGG

GGGCTCCGCGCACCAACAGGACTAGTTCTCAGGGCGCTTTGCTCCTTGTTTTTTACCCTC

GTTCTGTTTTCTCCCCTTCTCCGGAAGGCTTGTCAAGGGGTAGGAGAAAGAGACGCAAAC

ACAAAAGTGGAAAACAGTTAATGACCAGCCACGGCATCCCTGCTGTGAGCTCTGGCCGCT

GCCTTCCAGGGCTCCCGAGCCACACGCTGTGGGTGCTGGCTGAGGGAAC

>A1_Vervet_AGM

GGAGGAAGGCGAGAACAGGCTTTGACCGATAGTAACCTCTGCGCTCCGGGCAGCCGAATC

TATAAAAGGAACTAGTCTCGGCAAAAACCCCGTAATTGCGAGCGAGAGTGAGTGGGGCCG

GGACCCGCAGAGCCGAGCCGACCCTTCTCTCCCGGGCTGCGGCAGAGCAGGGCGGGGGGC

TCCGCGCACCAACAGGACTAGTTCTCAGGGCGCTTTGCTCCTTGTTTTTTCCCCTCCTTC

TGTTTTCTCCCCTTCTCCGGAAGGCTTGTCAAGGGGTAGGAGAAAGAGACGCAAACACAA

AAGTGGAAAACAGTTAATGACCAGCCACGGCGTCCCTGCTGTGAGCTCTGGCCGCTGCCT

TCCAGGGCTCCCGAGCCACACGCTGTGGGTGCTGGCTGAGGGAAC

>A1_Bolivian_s_monkey

GGAGGAGGGAGAGAACAGGCTTTGACCGATAGTAACCTCGGCGCTCGGTGCAGCCGAATC

TATAAAAGGAACTAGTCTCGGCAAAAACCCCGTAATTCCGAGGGAGAGTGAGTGGGGCCG

GGACCCGCAGAGCCGACCGACGCCTCTCTCCCCGGCTGCGGCAGCGCAGGGCAGGGCAGG

GTGGGGGGCGCCGCGCGCCTGCAGGGCCAGTTCTCAGGACGCTTTGCTGCTTGTTTTTTC

TCTGGTTCAGTTTTCTCCCCGTCTCCGGAAGGCTTGTCAAGGGGTGGGAGAAAGAGACGC

CAACACAAAAGTGGAAAACAGTTAATGACTAGCCACGGTGTCCCTGCTGTGAGCTCTGGC

CACTGCCTTCCAGGGCTCCTGAGCCACACACTGTGGGTGCTGGCTGAGGTAAC

>A1_Marmoset

GTAATTCCGAGGGAGAGTGAGTGGGGCCGGGACCCGCAGAGCCGAGCCGACGCCTCTCTC

CGGGGCTGCGGCAGCGCAGGGCAGGGCAGGGTGGGGGGCGCCGCGCGCCAACAGGGCCAG

TTCTCAGGACGCTTTGCTTCTTTTTTTCTCCGGTTCTGTTTTCTCCCCTTCTCTGGAAGG

CTTGTCAAGGGGTGGGAGAAAGAGACGCCAACACAAAAGTGGAAAACAGTTAATGACCAG

CCACGGCGTCCCTGCTGTGAGCTCTGGCCGCTGTCTTCCAGGGCTCCTGAGCCACACGCT

GTGGGTGCTGGCTGAGGTAAC

>A1_Tarsier

AGAGAACAGGCTTTGACCGATAGTAACCCCTGCGCTCAGTGCAGCCGAATCTATAAAAGG

AACTAGTCTAGGCAAAAACCCGTAATTCCGTGGGAGAGTGAGTGGGGCCGGGACCCGCCG

AGCCGAGCCGACATCTCTCTCCCTGGGAGCGGGTGGGCAGAGCAGAGGGTGCCCCACGCC

ACCAGGGTTAATTTCTCGGGGCGCTTTGCTCCCTGGTTTTTTTTTTTTTTTTTCCC

CTTTTCCCTTTTCTTTTTCTCCCCTTCACCGGAAGGGTTTGCCTAGGGGTGGGGGAGAGA

CGCAAACACAAAAGCAGAAAACAGTTAATGACCAGCCACTGTGTCCCTGCTGCGAGCTCT

GGCTACTGCCTTCCAGGGCTCCCAAGCCACACGCTGTGGGTGCTGGCGGAGGGAAC

>A1_Mouse_lemur

GGAGGAGGCAGAGAGCAGGCTCTGACCGATAGTAACCCCTGCGCTCAGTGCAGCCGAATC

TATAAAAGGAACTAGTCTCGGCAAAAACCCGGTAATTCCGAGAGAGACTGAGAGGGGCCG

GGACCCGCCGGGCCGGGTGGACACCGCTGTCTCCGGGCGCGGGTGGGCAGGGCGAGGGGG

CGCCGCAGGGCCAGTTTCTCGGGGCGCTCCGCTCCCTGTTTTTTTCTCTCACTTTTCCC

CCCTCCCCTTCTCGGGAAGGGCTTGTCAAGAGGTGAGGGGAAGAGACGCAAACACAAAAG

TGGAAAGCAGTTAATGACCAGCCACAGTATCCCTGCTGTGAGCTCTGGCCACTGCCTTCC

AGGGCTCCTGAGCCACACGCTGTGGGTGCCGGCTGAGGTGAC

>A1_Bushbaby

GACCAGCCACAGCATCCCTGCTGTGAGCTCTGGCCACTGTCTTCCAGGGCTCCTGAGGAG

CCAGACTCCGTGGGTGCCAGCGGAGGTGAC

>A1_Long_t_chinchilla

TACATAAACAGAGGCCGCGGAGGGGCGGGGGCGGAGGGAGAGAACAGGCTTTGACCGCTG

GTAACCCCTGCGCTCGGCGCAGCCGAATCTATAAAAGGAACTAGTCGCGGCAAAAACCAG

TGATTCCGAGGGAGAGTGCGAGGGGTCGGGACCTGCGGAGCCGAGCCGCACATCTGGTCC

GCGAGCCGGGCACGCAGGGCAGGGCAGGGCAGGGCAGGGCAGGGGGCGCCGCAGGACGCG

CGCAGCTAGAGCCAGTTCTCCGGGGCGCGCCGCCCCTTGCGTCCTCTCCCCGTTTTTGTT

TTTCCCCCTCCCCTTCACTGGAAGGGTTTGTCAAGGGGTGGGGGCAAGAAACGCAAACAC

AAAAGTGGAAAACAGTTAATGACCAGCCACGGTGTCCCTGCCGTGAGCTCTGGCCGCCGC

CTCCAGGGCTCCCAAGCCACACACAAGCTTCTGCGGTGTTGGGGGTGGTAAC

>A1_Degu

TTAATGACCAGCCACAGTGTCCCTGCTGTGAGCTCTGGCCACCGCCTCCAGAGCTCCCAA

GCCACACACAAGCTTCTGCGGCATTGGAGGAGGTAAC

>A1_Naked_mole_rat_m

ACTAGTCGCGGCAAAAACCAGTGATTCCGAGGGAGAGTGAGAGGGGCCGGGGGACCTGCA

GAGCCGAGCCCACTCTTCTGGTCCCCGAGCTGGGCAGGGGAGGGCAGGGGACGCCGCGAT

ACCCACGCACCTAGAGCCAGTTTCTCGGGGGCGCTTCGCTCCCTGCGCCCCCACCCCCTT

TTTTCCTCGCCTTCCTGGAAGGGTTTGTCAAGGGGTGGGCAAGAAACGCAAACGCAAAAG

AGGAAAACAGTTAATGACCAGCCACAGCGTCCCTGTTGTGAGCTCTGGCTGCTGCCTCCA

GGGCTCCCAAGCCACACACGGGCTTCTGTGGTGTTGGAGGAGGCAAC

>A1_Naked_mole_rat_f

ACTAGTCGCGGCAAAAACCAGTGATTCCGAGGGAGAGTGAGAGGGGCCGGGGGACCTGCA

GAGCCGAGCCCACTCTTCTGGTCCCCGAGCTGGGCAGGGGAGGGCAGGGGACGCCGCGAT

ACCCACGCACCTAGAGCCAGTTTCTCGGGGGCGCTTCGCTCCCTGCGCCCCCACCCCCTT

TTTTCCTCGCCTTCCTGGAAGGGTTTGTCAAGGGGTGGGCAAGAAACGCAAACGCAAAAG

AGGAAAACAGTTAATGACCAGCCACAGCGTCCCTGTTGTGAGCTCTGGCTGCTGCCTCCA

GGGCTCCCAAGCCACACACGGGCTTCTGTGGTGTTGGAGGAGGCAAC

>A1_Squirrel

GCTCGGCGCAGCCGAATCTATAAAAGGAACTAGTCGCGGCAAAACCCAGTAATTCCGAGG

GAGAGTGAGTGGGGCCGGGACCTGCCGAGCCGAGCCCACACCTCTCTTCCCCTACGTGCG

TGGGCAGGACAGGGGGCTCCACAGGACGGCGCAGCCGAAGCCCATTTCCAGGGGCGCTTT

GCTCCCCGTTTTCCCACACTTCTTTCTCTGCCATTCACCGGAACGGTTGGTCAAGGGGTG

GGGGAAAGAGACGCAAACACAAAAGTGGAAAACAGTTAATGACCAGCCACAGCGTCTGCT

GTGAGCTTCGGCCAGTGCCTCCAGGGCTCCTGAGCCACACACAGGTTGCTTTGGGTGCCG

GCTGAGGTAAC

>A1_Chinese_hamster_CG

TAACCCCGGTGCTCGGCACAGCCGAATCTATAAAAGGAACTAGTCGCGGCAAAAACCAGT

AATTCCGAGGGCGAGCGAGCGGGCCGGGACCGGCCGAGCCCACATCTCTCTACACAGCGC

AGGGCTGGGCAGGGGCGCCTCGGGACCCGCTCACCCACAGCCGGTTTGGGGCGCTGCTCT

GCTCCCTGTTTCCCTTCACTTTTCCCTCCCCCTTGCTGGAAGGGTTTGTGCAGGGTGGGC

TAAACAGACGCAAACACCAAAGTGGAAAACAGTTAATGACCAGCCACAGCGTCGCTGCTG

TGCACTCTGGCTGCTTCCTCCAGGGCTCTGGAGCCACACGCACGGGTTGCTGTGGGTTCC

AGCGGAGGTAAC

>A1_Golden_Hamster

GCGAGCGGGCCGGCACGGGCCGGGCCCACTTCTCTCCACCTAGCGCAGGGCTGGGCAGGG

GCGCCGCGGGGACCCGCTCGCCCACAGCCGGTTTGGGGCGCTGCTCTGCTCCCTGTTTCT

TCCTCTCCCCCCAAACACACACTTTTCCCTCCCCCTTTCTGGAAGGGTTTGTGCAGGGTG

GGAGAAACAGACGCCAACACCGAAGTGGCAAACAGTTAATGACCAGCCACAGTGCCGTGT

GCTCTGGCTGCTTCCTCCAGGGCTCTGGGGCCACACACACGGCTCGCCGTGGGTTCCAGC

AGACGCAAC

>A1_Prairie_vole

AGCAATTCCGAGGGCGAGCGAGCGGGCCGGGACCAGCCGAGCCCACACCTCTCTCCGCAG

CGCAGGGCTGGGCAGGGGCGCCGCGGGACCCGCTCAGCCACAGCCGGTTTGGGGCGCTGC

TCTGCTCCATTTCCCCCCACCCCACTTTTTCTCTCCCCCTTTCTGGAAGGGTTTGTGCAG

GGTGGGAGACACAGACGCAAACACCAAAGTGGAAGACAGTTAATGACCAGCCACAGCGTC

ACTGCTGCGCGCGCTGGTTGCTTCCTCCAGGGCTCTCGAGCCACATGCACAGGTTGCTCT

GGGTGCCAGCAGAGGTAAC

>A1_N_A_deer_mouse

AGTAATTCCGAGGGCGAGCGAGCGGGCCGCGACCGGCCAAGCCCACATCTCTCTCCGCAG

CGCAGGGCTGGGCAGGGGCGCCGCGGGACCCGCTCAGTCACAGCCGGTTTGGGGCGCTCC

TCTGCTCCCTGGTCCCCCCACTTTTTTCCCCCTCCCCCTTTCTGGAAGGGTTTGTGCAAG

GTGGGAGAACACAGACACAAACACCAAACTGGAAAACAGTTAATGACCAGCCACAGCGTC

ACTGCTGTGTGCTCTGGCTGCTTCCTCCAGGGCTCGCGAGCCACACACACAGGTCGCTGT

GGCTGCCAGCGGAGGTAAC

>A1_Mouse

AGTAATTCCGAGGGCGAGCGAGCGGGCCGGGACCGGCAGAGCCCACTTCTCTCCGCGGCG

CAGCGCAAAGCTGGGCAGGGGGCGCCGCGGGACCCGCGCAACCACAGCCGGCTTGGGGAG

CTGCTCTGCTCCCTGTTTCCCCCCACTTTTTTCTTCCCCTTTCTGGAAGGGTTTGTGCAG

GGGTAGGGAAAACAGACTCAAACAGCAAAGTGGAAAACAGTTAATGACCAGCCACAGAGT

CACAGCTCTGTGCTCTGGCTGCTCCCTCCAGGGCTCTCGAGCCGCAGACGCAGGTCGCTG

TGGGTGCCGGCTGTGGTGAC

>A1_Algerian_mouse

AGTAATTCCGAGGGCGAGCGAGCGGGCCGGGACCGGCAGAGCCCACTTCTCTCCGCGGCG

CAGCGCAAAGCTGGGCAGGGGGCGCCGCGGGACCCGCGCAGCCACAGCCGGCTTGGGGAG

CTGCTCTTCTCCCTGTTCCCCCCCCCCCCACTTTTTTCTCCCCCTTTCTGGAAGGGTTTG

TGCAGGGGTAGGGAAAACAGTCTCAAACAGCAAAGTGGAAAACAGTTAATGACCAGCCAC

AGAGTCACAACTCTGTGCTCTGGCTGCTCCCTCCAGGGCTCTCGAGCCACAGACGCAGGT

CGCTGTGGGTGCCGGCTGCGGTGAC

>A1_Ryukyu_mouse

AGTAATTCCGAGGGCGAGCGAGCGGGCCGGGACCGGCAGAACCCACTTCTCTCCGCGGCG

CAGCGCAAAGCTGGGCAGGGGGCGCCGCGGGACCCGCGCAGCCACAGCCGGCTTGCTCCG

TGTTTCCCCCCACTTTTTTCTCCCCCTTTCTGGAAGGGTTTGTGCAGGGGTAGGGAAAAC

AGACTCAGACAGCAAAGTGGAAAACAGTTAATGACCAGCCACAGAGTCACAGCTCTGTGC

TCTGGCTGCTCCCTTCAGGGCTCTCGAGCCACAGACGCAGGTCGCTGTGGGTGCCGGCTG

CGGTGAC

>A1_Shrew_mouse

AGTAATTCCGAGGGCGAGCGAGCGGGCCGGGACCGGCAGAGCCCACTTCTCTCCGCGGCG

CAGCGCAAAGCTGGGCAGGGGCCGCCGCGGGACCCGCGCAGCCATAGCCGGCTCGGGAAG

CTGCTCTGCTCCCTGTTTTTCCCCACTTTTTTCTCCCCCTTTCTGGAAGGGTTTGTGTAG

GGGTGGGGAAAACAAGACTCAAACAGCAAAGTGGAAAACAGTTAATGACCAGCCACGGAG

TCACAGCTCTGTGCTCTGGCTGCTTCCTCCAGGGCTCTCAAGCCACACGCGCAGGTTGCT

GTGGGTGCCGGCTGCGGTGAC

>A1_Rat

TAACCCCGGCGCTCGGCACAGCCGAATCTATAAAAGGAACTAGTCGCGGCAAAAACCAGT

AATTCCGAGAGCGAGCGAGCGGGCCGGGACCGGCAGAGCCCACTTCTGTCTCTGCGGCTC

AGCGCAAAGCTGGGCAGGGGGCGCCGCGGGACCCGCGCAACCACAGCCGGCTTGGGGAGC

TGCTCTGCTCCCTGTCCCCACCCCCTACTTTTTTCTCCCGGTTTCTGGAAGGGTTTGTGT

AGGGGTTGGGTAAACAGACGCAAACAGCAAAGTGGAAAACAGTTAATGACCAGCCACAGT

GTCACAGCTCCGTGCTCTGGCTGCTTCCTCCAGGGCTCTCAAGCCACACACACAGATCAC

TGTGGGTGCTGGCAGAGGTAAC

>A1_U_G_m_b_mole_rat

GGCGAGCGAGCGGGCCGAAGCTGCGCAGGGCAGGGGCACGCCGACCCGCAACCACAGGCA

CTCTGGGGGTGCTCCACTCCCCGCCCCCACCCCCGCTTTCTTTCTCTCCCCTTCTCTAGA

AGGGTTTGTCCAGGGGTGGGGAAAACAGACGCAAACATCAAAGTGGAAAACAGTTAATGA

CCAGCCAAAGCGTCCCTGCTGTGAGCTCTGGCCACTGCCTCCAGAGCTCCTGAGCCACAC

ACGCAGGTCACCGTGGGTGCCAGCAGAGGTAAC

>A1_Lesser_E_jerboa

CAGTTAATGACCAGCCACAGCGTCCCTGCTGTGAGCTCTGGCCACTGCCTCCAGGCCTGT

GGAGCCACAAGCAGGACAGTGTGAAGGGTCAGCAGAAGAAAC

>A1_Rabbit

AGGAGAGAACAGGCTTTGACCGATAGTAACCCCCCGGCGCCCGGAACGCCCAATTCTTTA

AAAGGAACTAGTCGCGGAAAAAAACAGTTATTTCCGAACGAAGATGAGTTGCGGTCGGAA

CGGCCCAGGCCGCGCCGACCCCCTTCTTCCCAAGGCGCGGGCGGACAGGGTTGGGGGCCC

CCAGGACCCCGGACAGCCAGGGCCTATTGCTTCCTGCGCTTTGCTCCCTAATTTTTTTTT

GCCCCCTCTTCTTTTCCTCCCCTTCTCCGGAAGGGTTTGTCACGGGGTTGGGGGAAAGAG

GCGCAAACAAAAGTGGAAAGCAGTTAATGACCAGCCACAGCGGCTTTGCTACAAGCTCTG

GCCGCTGCCTCCAAGGGCGAGCCACAAGCAGGTTGCCGTGGGTGCCTGCTGAGGTAAC

>A1_Ferret

GGTTTTTCAAGGGGTTGGGGACAAAGACGCACGCACAAAAGTGGAAAACAGTTAATGACC

AGCCACAGCGGCCCTGCCGTGAGTTGCGGCCACTGCCTCCACGGGCTCCTGAGCCACACG

CATCTGCTGGGGGTGCTAGCAGCGGTAAC

>A1_Dog

TTAATGACCAGCCACGGCGGCCGCCTCCACGGGCCCCCGAGCCACACGCACGGTGCTGGC

GCCGCCCGCTGAGCCGAC

>A1_Cat

GAACAGGCTTTGACCGATAGTAACCCTGGCGCTCAGCGCAGCCGAATCTATAAAAGGAAC

TAGTCGCGGCAAAACCCCGGTAATTCCGAGGGAGAGTGAGTGGCGCCGGGATCCGCCGAG

CCCAGCGGACACCTCTCTCCTCGGGCGCAGGTGGGCAGGGCAGGGGGGGCGCCGCTGGAC

CGGCGCTGCCAGAACCAATTTTTCGGGGCGCTTTGCTCCTCTTTTTTCCCCCTTTTCCCT

TCTCCGGAAGGGTTTTTCAAGGGGTGGGGGAAAAGACGCACACACAAAAGTGGAAAACAG

TTAATGACCAGCCACAGCGTCCCTGCTGTGAGCTGCTGCTGCTGCCTCCAGAAGCTCCTG

AGCCACACACAGGTTGCTGCGGCTGCCGGCTGAGGAAAC

>A1_Horse

GTAATTCCGAGAGAGAGTGAGTGGGGCTGGCACCGGCCGAGCCGAGCTGACCCCTGTCCG

CCCGGGCGCAGGTGGGCAGGGCCGGGGCCCCGCGGGACCAGCGCCACCAGAGCCAATTTC

GCGGCGCGCTTTGCTCCTTTTTCCCCCTTTTTTACCGGAAGGGATTTTCAAAGGGTGGGG

GAAAGACAGGCAAACACAAAAGTGGAAAACAGTTAATGACCAGCCACAGCGTCCCTGCCC

TGAGCTCTGGCCACGGCCTTCACAGGCTCCCAAGCCACACGCAGGTTGCCGTGGGTGCCA

GCTGAGGTAAC

>A1_Cow

AAGAGGGTGGCCTATTGACTGGCCATGACTGTGAAGTGGGACGCTTTCCAACCAGTAACT

GGAAATGTGCACAGGGGCAGAAATGAGTGTGCTACTAGCTTTGAAGCATTGAGAAATCTA

GTCAATACATAGTACGGCCTTCTTTTTGGTTTTGGTTTGTTTGTTTGCTTCCTTGGAAAA

CTGATGTAGAAATGTCCCTTTGGCCCCAGTTACCTGAGAAGAGGATTTGGGCATTGCCAA

AACCTCCTCTTTGGATAAAATTGCTCCCAGCAGTGGTCACCATGTTCTGAGGTGGGAGGA

GGGAATTATGCAAGAGGCCCAGGGCTATGCCAAGGTGGGTAGGAAAGCGGGGCAGGTACC

CAGCGAAGCCGGAGCGATACCTTCCACCGCTGCATAGGCTACTTTTGCTGACGTCTCTGC

GTGTCTCCTGGAGGGTCTCTGAAGCCAGGATCTGTGTCTGGAAATGCAGAAACCCCGCTT

TAGGAAAACACTCTCCAGAAAGCTTCCTCCTGGCCGTTCTCGTCTCGCGCCCATTCTGCA

GAGTTAATGACCAGCCACAGCGTCCCTGCTGTGAGCTCCAGCCACTTCCTTCCCGGGCTC

CCGAGCCTCACGCAGGCTGCTGTGGTGCTGGCTGAGGTAAC

>A1_Sheep

CTCTCTACCCCGGCGCAGGTGGGCAGAACAGGGGGCGCCGTGCAACCAGCGCCGCCACAG

CCAATTTCTCGGGGCGCTTTGCTCCCTTTACCCCCCTTCTTTTCCGAAGGGTTTTTCAAG

GGGTTGGGGAAAGAGCCACAAACACAAAAGTGGAAAACAGTTAATGACCAGCCACAGCGT

CCCTGCTGTGAGCTCCAGCCACTTCCTTCCCAGGCTCCCGAGCCTCACGCAGGCTGCTGT

GGTGCTGGCTGAGGTAAC

>A1_Armadillo

GCCGAGCCCAGGCTTCGACCGATAGTAACCCCGGCGCCACCGAATCTATAAAAGGAACTA

GTCGCGGCAAAACCCCGGTAATTCCAAGCCAGAGTGAGTGGGGCCCCGATCGGCAGAGGC

CAGCCGACGCGTCGCTGCCCCGGTGCGGGTGGGCTGGGCAGGGGGCGTCGCAGGACCCGC

GCCGCCGCCCGGGCCAGTTCCACGAGGCTTTGCCCATTGTTTTTCTGCTTTTCCCCAGTT

TCCAGAAGGGTTCAGTCGAGGGGTGGGGGAAAGAGTCGCAGACACCAAAGTGGAAAACAG

TTAATGACCAGCCACAGCGTCCCTGCCGTGAGTTCCGGCCACTGCCCCCACGGGCTCCCG

ACGAGCCACACGCAGGTTGCCTGGGCACGGCTGAGGTAAC

>A1_Tasmanian_devil

TTAATGACCAGCCACAGTGTCCCTGCCCAGCGCTACCTCTGCTCCCTCCACTATCTGCCT

GGCTGAAGGGAGCTGTGGGTGTATGCTGAGGAAAG

>A1_Opossum

GAAAGAGAACAGAGTTTGACCGATAATAACCCCGCTGTTCAGCGCAGCCGAATCTATAAA

AGGAACTAGTCCAGGCTAAAAACCCTTAATTCTGGGAGAGAGGGACTGAGGACCATATGC

TAGGCTGTGCAGGAGGAGGCCGTTGGTGCCCACAGGAAAGCCTAGGCAGGGGTTGGCAAA

GGCAAGGGAGGGGGTCACCAAAGGGGTATCGGAGGGGCGAGAGCCCGTTTGTCGGCTCTC

TCTCTCTCTCTCTCTTTTTTTTTTTTGTTTTCCGCAAGTATTTCCAAAGGGGTAGGGGAG

AGAGTCCCAGAAACAAAAGTGGAAAGCAGTTAATGACCAGCCACGGTGTCCTTGCCCAGT

GCTGCCTCTGCTCCCTCCACTATCTGCTGGACTGAGGGGAGCTGTGGGTGCGTGCTGAGG

AGAG

>A1_Platypus

TGCTGTAATGACTAATGAGAACTCTCCTCCTCTCCTGTTTTATCTTCCAGTTAATGACCA

GCCACAGTGTCCCTGCAGTGTGCTGTTGCCACTGCCCCTGTCACACCAGGGGAGCTGTGC

CTGCATGCTGAGGAAAG

>A1_Chicken

CCGGGAGCGCGGCCGGCGTCGGGGCCGGATCCGTGAGTGGCAGAGCTGCGCTCGGGAGAA

GTGGAGCGAAGCGAGCCGACTCGGCTCGGTGCGGGACCGAGGGCGGCTGCCCAGGGCTCC

CCGGCCCGGAGTTAATGCCCAGCCATAACGTCCTTGTTGTGTACTGCTGCTGCTGCACAA

AGGGAAGAAGGCATTGCTGAAGAACG

>A1_Duck

AGTTAATGCCCAGCCACAACCTCCTTGCTGTATACTGCTGCTGCTGCACAAGAGGAGCAA

GGCGTTGCTGAAGAGCG

>A1_Flycatcher

TTAATGCCTGGCCACAACATCTGTACTGTACTGCTGCTGCTACACAAAGAGAGCTTCTAG

TCTTTGCTGAAGGAAG

>A1_Anole_lizard

GGCAGAAAGGAAGCCGCGCTTGACCGATAATAACCTCCGCGGCGCAGCGCCGGCGGGATC

TATATAAGGCGCTCCGAGTGCGGGACTGGCCCTGGTTTGGCCCCAGCCTCGTCGGTGGGA

GCCACAAAGGACGACCGGAGAAGCCCTCCATCGACTGGCTCTCCGATCTACTCCGGGAGG

GAGAGAAAGATCGCCTCCCCATCAAACTGGCGAGGAGTTGATGACCAGCCACAGTAGCTC

TGCTGTGTGCTGTTTCCATCCAAGGTGCTGATTGCTGGTACATACAGAAGGAAG

>A1_Coelacanth

GCCTGTACAGCGCTGCTCGGTGTGCACTATAAAAGCAACTAGAGGTTAAAAAAAAAACAT

TCAGTCTTACATCGAATCAGAGACACCTCAAGACCATATATATCGTTCACAGTAAACAAA

GAAAAGAAAAAATATATATATTTAAGGATTTTAAAGAAAGATACTATATATTTCCTAGTA

GGGATTTGTCATTTTTCTGGTTTAACTATATTTTACATATACAGCCAAGTGGGGAAAAAA

GTTAATGTCCGACAACAATATCCCTGCTGCATGAGTGGAGCTGCTACTGGGAAAAG

>A1a_Platyfish

TGACCGTTAGTTACCTTTTGCTGCGCTCCGCGTCGAGCTGCGTATAAAAGCCTGCCTGGG

GAGAAAAAACAGTTTTCCTAGACACTGACAAAGAGCAAGGAGGACCGCGGCGTCAGAGGG

ACGCAGTTGTTAACCTCGTTTTTCGCTTTTGTTTGAGAAAAGGACGTTCAATTGATGAGA

GGCAGAAGAGCGAGTAAACACATTAAGATCAAGTGAGGAGCTGGCGTTGGCTTGTTGAGG

TTAGGAAGACGGCAAC

>A1a_Medaka

GCGAGACTCACCGCGAGTCCCGACAAGGGAAGTTTATTCCTCGTTTTTCTGGGACACCCT

ATGACACGAACAAGAGTGGATAGAAAAAAGTGAGGATCAGGAGTTTGCTTGTGAAGGTGG

GGAGAAGACAAC

>A1a_Tilapia

TGAGGAGCTGGAGCGTGCTCAGTGAGGTTGGGAAGACGGCAAC

>A1a_Tetraodon

TCTAGCCCGTTTACTGAGCGTCTTTTCTCTGTCAACTTTGCTTCATTAGTGAGGAGCTGG

AGCGTGCTCGGTGAGGTTGGGACGACATCAAC

>A1a_Zebrafish

GGAAAAAGGTCCGTCCATAAGAACACAAAGCACCACCAGTACAATATCGCTCGGAGAGAG

TTTAAACAGCTGCTTTATCCTCTCTTTTCTAAAAGGATTTACAGTTTATATGAAATAAGG

AACAATCATTAAGGAGCAAGAAATAGCGCTTCTGTAAGGCGGCACGGATAGTGTGTATCC

GTGGTAAG

>A1b_Tilapia

ACCGGTTTGTAAAACTTCCACCAACATTCTTGTAGTTCGTTTCCTCCCTGGCGGTCGGGT

TTTATTTTGAAGGTCCTGGCGGAAGTGTGTTGTGACTATTGGGGCTTTACTCCGGTGAGT

TTTGTCCGGCGGCTGCTGCTCCGTGAGCAGCAG

>A1b_Cave_fish

TTTATTTATTCATTTTACTCCAACATATACCTATAAATAGAAAAATCATAGAAAATCAGA

GCTGTATAATAACTATATCAAGACAGAAAGAACAAAATGGAGCTGCCAACATTGTATTCT

TTAATTTTACATTTTCACTCATTCATCCCTAACTATTTCTGGCATAGTCCTCACACTTTC

TTTGATCTCCAAATCAATTCTCTTCTTCTATTTCCTTAATTTTCCCATTTTTCCTAGTTT

TTCGGCATGTGATTGAATCAGTGTAAAGTCATTTGGCCACATTAACTTTGAACAGTGATG

GCTTGACCGTAAGACCACACACAAGCCCTCGGCCCATATGAATAGTTTCATTAGCATAGA

ACAATGGCGACAGATTGACTCTCAGACTGCCATTGAAGAGCTTTGATCAAAAGCTGCCGT

TCCTGGAGCAGCTACCTGGAGCTGTGAAATTCCTTACAGAACGATTTACCAGACTACAGT

GAAGTCCATTATTATTACTATTATAAAAGAAGAGAGGGGTGGGTGTTCCTGAGTGAGATG

AGTCAGGACACAACAGGACAGGAGAAGACAGGAGGACGGGGGAAACGTGAGCAGAACGGA

TGCAGATAGGCTGGTGCACTTTGTCCTGGACCTTTGAGCTGCTCTGTTGGTTGCACTTGG

AATCCTTCTTTTACTCAGCCTGTGCTCTCTCTGTGTCTCACTGTCAGCTGGAGAGCGCAG

CGTGAGGAGGACCTGAGTGAAG

>A1b1_Zebrafish

GACCGACAGTAACCCAGGCGCTCCGAGCGGATGCGCTATAAAACCACTTTAAACCCTTCA

GATGCCCCTTCACACGAGAACCACCGGCGTCAGACTCCACAAGGACGAATAAACATCTGA

TTCACTTGGATTACAACGAATGTCCTACACAAGTCATCAGCTGTGTCTGGTTGAGAGCTG

AGCGCGAAGAGGGACCCGAGTGAAG

>A1b2_Zebrafish

GAATAGCACCATTAGCATAGAGCAAGATCTCGACTTCCAGCAAACCTGTTCATTCGAAAT

GATCAAACCATTAAGAGACTCTAACGGCCTGCGAAATTCCTTTCTTGATGAATCTCTGAC

ACTACAAGGTCCACCACTTCCAGGAATTCTGGAAAAAAAAAAAGACAGGAGATGAAAAGGG

AAGACGTGTGAGAGAGCAAGCGAACGCTGGAGAGTAAGATAGGAGCTGCTGCACTTTGTC

CTGGACTTTTGAGCTGCTCTGTTGGTCGCTGCTGGATGCTTTTTTGTCAGTCAGCCCCGT

CTCCGTTGTGTCATTGCAGTTGAGAGCTGAGCGCGAAGAGGGACCCGAGTGAAG

>A1a_Spotted_gar

CTGGACTCGAAGCCTGCACTTCCCTCACAGACTTCGTTATTGCGTGGCCCCTTACCTTAA

TAAAATCGATTTTAAATGCTCATAGATCCTCACATATGCCTTGGGAAATTTGTTTCCATC

TATAGGAGTTAAATCCTGTTTTCAACGCTCGGATTCTTAATTTTCATTTGGGACCTCTTT

GATTGTGAGGAATAAAACTCACATTTCTTGAGCGTTCATAGCTTAATTTTCTCTTGCGAA

ACGAATAGTTTTTTCTTTATTTGTCGCGCTGGAAGGCAGAAGGACAAAACACAATGGCAG

GGTAGCCCTTTTAAGAGCTGTTTTGACTCTACACGTGCTTCGCTTGCAATGACTGAGCCA

CACAAACACAGCGGAGACTGGGATCTACTCGACCGAGAGTAACCCTGCCTCCTCAGCGCA

GTGTACAGAGAGCGCTATAAAAACAGCTAAAGGTTAAAAACGGCAGTTTCACAGACCGAG

ACCGCACGGTTCACAGCGCAGAGAGAAGATACAACAAAAAGGAATCTTTTGCTTCGTTTA

CTTTAAAGGATTTAGCATTTATGCATAAAGACACATAAAGACTGCGCGAGATAATAGTCG

AGCGCCGGAGTCTGTGAGCTGGAACTCCTGAGGAAAG

Supplemental Table S1: Multi-FASTA format file containing *ABCA1* 5’UTR sequences of 55 species (59 transcripts without intron sequences) downloaded from the Ensembl database and evaluated within comparative analyses.

Supplemental Table S2

Table S2: Summary of 59 transcripts from Ensembl and comparison of 5'UTR sections

Sp. No.	Sp. Name	Transcript			Lenght of 5'UTR sections (bp)				Length of protein (aa)
		No.	Name	ID	Before intron	After intron	Whole 5'UTR	Intron	
1	Chimpanzee (Pan troglodytes)	1	ABCA1-201	ENSPTRT00000039236.5	320	92	412	31889	2261
2	Bonobo (Pan paniscus)	2	ABCA1-201	ENSPPAT00000058048.1	312	92	404	24206	2102
3	Human (Homo sapiens)	3	ABCA1-202	ENST00000374736.7	303	92	395	24163	2261
4	Gorilla (Gorilla gorilla)	4	ABCA1-201	ENSGGOT00000009477.3	312	92	404	24188	2261
5	Golden snub-nosed monkey (Rhinopithecus roxellana)	5		ENSRROT00000053936.1	323	92	415	26077	2261
6	Black snub-nosed monkey (Rhinopithecus bieti)	6	ABCA1-201	ENSRBIT00000047284.1	360	92	452	25732	2261
7	Angola colobus (Colobus angolensis palliatus)	7	ABCA1-201	ENSCANT00000035942.1	323	92	415	25552	2261
8	Crab-eating macaque (Macaca fascicularis)	8	ABCA1-201	ENSMFAT00000023951.1	715	92	807	24586	2261
9	Pig-tailed macaque (Macaca nemestrina)	9	ABCA1-201	ENSMNET00000039779.1	425	92	517	24876	2261
10	Macaque (Macaca mulatta)	10	ABCA1-201	ENSMMUT00000028999.3	360	92	452	24582	2261
11	Drill (Mandrillus leucophaeus)	11		ENSMLET00000035934.1	317	92	409	31834	1919
12	Vervet-AGM (Chlorocebus sabaeus)	12	ABCA1-201	ENSCSAT00000009382.1	313	92	405	24875	2261
13	Bolivian squirrel monkey (Saimiri boliviensis boliviensis)	13	ABCA1-201	ENSSBOT00000055395.1	321	92	413	26130	2261
14	Marmoset (Callithrix jacchus)	14	ABCA1-201	ENSCJAT00000032583.2	229	92	321	25833	2261
15	Tarsier (Carlito syrichta)	15	ABCA1-201	ENSTSYT00000031652.1	324	92	416	22034	2261
16	Mouse Lemur (Microcebus murinus)	16	ABCA1-201	ENSMICT00000063500.1	310	92	402	20008	2261
17	Bushbaby (Otolemur garnettii)	17	ABCA1-201	ENSOGAT00000009127.2	0	90	90	0	2264
18	Long-tailed chinchilla (Chinchilla lanigera)	18	ABCA1-201	ENSCLAT00000022302.1	375	97	472	22789	2260
19	Degu (Octodon degus)	19	ABCA1-201	ENSODET00000008072.1	0	97	97	0	2260
20	Naked mole-rat male (Heterocephalus glaber male)	20	ABCA1-201	ENSHGLT00100013811.1	250	97	347	16484	2260
20	Naked mole-rat female (Heterocephalus glaber female)	21	ABCA1-201	ENSHGLT00000016174.1	250	97	347	16244	2260
21	Squirrel (Ictidomys tridecemlineatus)	22	ABCA1-201	ENSSTOT00000001269.3	275	96	371	20032	2261
22	Chinese hamster CriGri (Cricetulus griseus crigri)	23	Abca1-201	ENSCGRT00000004943.1	272	100	372	16750	2261
23	Golden Hamster (Mesocricetus auratus)	24	Abca1-201	ENSMAUT00000018446.1	215	94	309	17080	2261
24	Prairie vole (Microtus ochrogaster)	25	Abca1-201	ENSMOCT00000016062.1	219	100	319	17510	2261
25	Northern American deer mouse (Peromyscus maniculatus bairdii)	26	Abca1-201	ENSPEMT00000010703.1	219	100	319	16816	2260
26	Mouse (Mus musculus)	27	Abca1-201	ENSMUST00000030010.3	220	100	320	15580	2261
27	Algerian mouse (Mus spretus)	28		MGP_SPRETEiJ_T0064864.1	225	100	325	16109	2261
28	Ryukyu mouse (Mus caroli)	29	Abca1-201	MGP_CAROLIEiJ_T0062396.1	207	100	307	16091	2277
29	Shrew mouse (Mus pahari)	30	Abca1-201	MGP_PahariEiJ_T0077533.1	221	100	321	16933	2261
30	Rat (Rattus norvegicus)	31	Abca1-201	ENSRNOT00000024564.7	282	100	382	15826	2261
31	Upper Galilee mountains blind mole rat (Nannospalax galili)	32	Abca1-201	ENSNGAT00000004944.1	173	100	273	24815	2261
32	Lesser Egyptian jerboa (Jaculus jaculus)	33	Abca1-201	ENSJJAT00000027060.1	0	102	102	0	2261
33	Rabbit (Oryctolagus cuniculus)	34	ABCA1-201	ENSOCUT00000009288.3	323	95	418	24629	2261
34	Ferret (Mustela putorius furo)	35	ABCA1-201	ENSMPUT00000007173.1	51	98	149	20857	2261
35	Dog (Canis familiaris)	36	ABCA1-201	ENSCAFT00000004346.3	0	78	78	0	2262
36	Cat (Felis catus)	37	ABCA1-201	ENSFCAT00000004356.4	300	99	399	20563	2261
37	Horse (Equus caballus)	38	ABCA1-201	ENSECAT00000011043.1	212	99	311	20272	2261
38	Cow (Bos taurus)	39	ABCA1-201	ENSBTAT00000027538.3	543	98	641	2481	2261
39	Sheep(Ovis aries)	40	ABCA1-201	ENSOART00000008003.1	160	98	258	19661	2261
40	Armadillo (Dasypus novemcinctus)	41	ABCA1-201	ENSDNOT00000009223.3	300	100	400	20170	2264
41	Tasmanian devil (Sarcophilus harrisii)	42	ABCA1-201	ENSSHAT00000008717.1	0	95	95	0	2264
42	Opossum (Monodelphis domestica)	43	ABCA1-201	ENSMODT00000020693.2	329	95	424	25789	2260
43	Platypus (Ornithorhynchus anatinus)	44	ABCA1-201	ENSOANT00000004496.2	0	137	137	0	2266
44	Chicken (Gallus gallus)	45	ABCA1-201	ENSGALT00000024891.7	131	75	206	15067	2260
45	Duck (Anas platyrhynchos)	46	ABCA1-201	ENSAPLT00000015088.1	0	77	77	0	2261
46	Flycatcher (Ficedula albicollis)	47	ABCA1-201	ENSFALT00000005231.1	0	76	76	0	2262
47	Anole lizard (Anolis carolinensis)	48	ABCA1-201	ENSACAT00000014788.2	217	77	294	35441	2258
48	Coelacanth (Latimeria chalumnae)	49	ABCA1-201	ENSLACT00000009960.1	241	55	296	33177	1525
49	Platyfish (Xiphophorus maculatus)	50	abca1a-201	ENSXMAT00000006594.1	213	43	256	3530	2270
50	Medaka (Oryzias latipes)	51	abca1a-201	ENSORLT00000017693.1	90	42	132	2863	2283
51	Tilapia (Oreochromis niloticus)	52	abca1a-201	ENSONIT00000020384.1	0	43	43	0	2269
52	Tetraodon (Tetraodon nigroviridis)	53	abca1a-201	ENSTNIT00000001913.1	49	43	92	119	2269
53	Zebrafish (Danio rerio)	54	abca1a-203	ENSDART00000167514.2	148	40	188	2551	2268
51	Tilapia (Oreochromis niloticus)	55	abca1b-201	ENSONIT00000002004.1	0	153	153	0	2337
54	Cave fish (Astyanax mexicanus)	56	abca1b-201	ENSAMXT00000016919.1	0	742	742	0	2293
53	Zebrafish (Danio rerio)	57	abca1b-201	ENSDART00000051556.5	170	35	205	5575	2282
53	Zebrafish (Danio rerio)	58	abca1b-202	ENSDART00000148461.2	0	354	354	0	2271
55	Spotted gar (Lepisosteus oculatus)	59	abca1a-201	ENSLOCT00000014184.1	597	40	637	3435	2262
	**Primates (N=17)**			**Median:**	**320**	**92**	**412**	**24875**	**2261**
	**Rodents (N=16)**			**Median:**	**221**	**100**	**321**	**16750**	**2261**
	**Placental mammals (N=7)**			**Median:**	**212**	**98**	**311**	**20170**	**2261**
	**Marsupials and Platypus (N=3)**			**Median:**	**0**	**95**	**137**	**0**	**2264**
	**Reptiles and Birds and Coelacanth (N=5)**			**Median:**	**131**	**76**	**206**	**15067**	**2260**
	**Ray finned fishes - abca1a (N=6)**			**Median:**	**119**	**43**	**160**	**2707**	**2269**
	**Ray finned fishes abca1b (N=3)**			**Median:**	**0**	**254**	**280**	**0**	**2288**

Abbreviations: sp., species;

Supplemental Table S2: Summary of all species and names and IDs of transcripts downloaded from the Ensembl database, lengths of 5'UTR sections evaluated - whole 5'UTR, 5'UTR section located before-Intron-1, section located after-Intron-1, whole Intron 1 lengths, and ABCA1 protein lengths in amino acids.

Supplemental Table S3

Table S3: Summary of alignment analyses (59 transcripts from Ensembl) performed with the help of 9 alignment programs.

Program	No. of most cons. nts	No. of most cons. nts within after Intron 1 sec.	Consensus seq. - start of 5'UTR sec. after Intron 1	Consensus seq. - end of 5'UTR sec. after Intron 1 preceding mORF
Clustal	40	38	AG↓TTAATGACCAGCCAC	GCTGAGGTAAC(ATG)
ClustalO	16	16	AG↓TTAATGACCAGCCAC	GCGGGGGTAAC(ATG)
GLprobs	37	37	AG↓TTAATGACCAGCCAC	GCTGAGGTAAC(ATG)
Mafft	30	28	AG↓TTAATGACCAGCCAC	GCTGAGGTAAC(ATG)
MSAprobs	38	38	AG↓TTAATGACCAGCCAC	GCTGAGGTAAC(ATG)
Muscle	41	39	AG↓TTAATGACCAGCCAC	GCTGAGGTAAC(ATG)
Probcons	39	39	AG↓TTAATGACCAGCCAC	GCTGAGGTAAC(ATG)
TCoffee	22	21	AG↓TTAATGACCAGCCAC	GCTGAGGTAAC(ATG)
WebPRANK	26	26	AG↓TTAATGACCAGCCAC	GCTGAGGGAAC(ATG)
**Median**	**37**	**37**		
**Range**	**16-41**	**16-39**		

Notes: The most conserved nts had >80% identity and >84% occupancy scores. Position of the Intron 1 was specified by an arrow sign. ATG sequence in brackets is the first codon of the mORF.

Abbreviations: cons., conserved; no, number; nts, nucleotides; sec., section; seq., sequence;

Supplemental Table S3: Summary of multiple-sequence (59 transcripts) alignment analyses performed with the help of 9 alignment programs. Highly conserved nucleotides are those with >80% identity and >84% occupancy scores.

Supplemental Table S4

Table S4: Upstream ATG and non-ATG start as well as stop codon comparison

			No. of upstream start codons										No. of upstream stop codons					
			uATGs		uGTGs		uCTGs		uTTGs		uACGs		uTAGs		uTAAs		uTGAs	
**Sp. No.**	**Sp. Name**	**Tr. No.**	**Wh. 5'UTR**	**Af. In. 1 section**	**Wh. 5'UTR**	**Af. In. 1 section**	**Wh. 5'UTR**	**Af. In. 1 section**	**Wh. 5'UTR**	**Af. In. 1 section**	**Wh. 5'UTR**	**Af. In. 1 section**	**Wh. 5'UTR**	**Af. In. 1 section**	**Wh. 5'UTR**	**Af. In. 1 section**	**Wh. 5'UTR**	**Af. In. 1 section**
**1**	Chimpanzee (Pan troglodytes)	1	1	1	7	3	10	7	5	0	4	2	3	0	4	1	5	3
**2**	Bonobo (Pan paniscus)	2	1	1	7	3	10	7	5	0	4	2	3	0	4	1	5	3
**3**	Human (Homo sapiens)	3	1	1	6	2	10	7	5	0	3	2	3	0	4	1	5	3
**4**	Gorilla (Gorilla gorilla)	4	1	1	6	3	9	7	6	0	3	2	3	0	4	1	5	3
**5**	Golden snub-nosed monkey (Rhinopithecus roxellana)	5	1	1	6	3	10	7	5	0	2	1	3	0	4	1	5	3
**6**	Black snub-nosed monkey (Rhinopithecus bieti)	6	1	1	6	3	11	7	5	0	2	1	3	0	5	1	6	3
**7**	Angola colobus (Colobus angolensis palliatus)	7	1	1	6	3	13	7	5	0	3	2	4	0	4	1	5	3
**8**	Crab-eating macaque (Macaca fascicularis)	8	3	1	9	3	21	8	8	0	6	2	9	0	7	1	13	4
**9**	Pig-tailed macaque (Macaca nemestrina)	9	1	1	9	3	15	8	6	0	5	2	4	0	6	1	11	4
**10**	Macaque (Macaca mulatta)	10	1	1	6	3	12	8	6	0	3	2	4	0	5	1	7	4
**11**	Drill (Mandrillus leucophaeus)	11	1	1	6	3	9	7	6	0	3	2	4	0	4	1	5	3
**12**	Vervet-AGM (Chlorocebus sabaeus)	12	1	1	6	3	10	7	5	0	3	2	4	0	4	1	5	3
**13**	Bolivian squirrel monkey (Saimiri boliviensis boliviensis)	13	1	1	10	4	12	8	4	0	4	1	3	1	5	2	6	4
**14**	Marmoset (Callithrix jacchus)	14	1	1	8	3	11	8	2	0	5	2	0	0	3	2	5	4
**15**	Tarsier (Carlito syrichta)	15	1	1	10	3	10	7	3	0	3	1	4	0	5	1	3	1
**16**	Mouse Lemur (Microcebus murinus)	16	1	1	9	4	12	7	1	0	2	1	2	0	4	1	8	5
**17**	Bushbaby (Otolemur garnettii)	17	0	nr.	4	nr.	5	nr.	0	nr.	0	nr.	0	nr.	0	nr.	3	nr.
**18**	Long-tailed chinchilla (Chinchilla lanigera)	18	1	1	8	4	8	3	5	1	4	1	2	0	5	2	4	2
**19**	Degu (Octodon degus)	19	1	nr.	2	nr.	4	nr.	1	nr.	0	nr.	0	nr.	2	nr.	2	nr.
**20**	Naked mole-rat male (Heterocephalus glaber male)	20	1	1	6	3	10	5	3	2	5	1	2	0	1	1	4	2
**20**	Naked mole-rat female (Heterocephalus glaber female)	21	1	1	6	3	10	5	3	2	5	1	2	0	1	1	4	2
**21**	Squirrel (Ictidomys tridecemlineatus)	22	1	1	9	3	6	4	4	2	4	0	1	0	4	2	5	4
**22**	Chinese hamster CriGri (Cricetulus griseus crigri)	23	1	1	6	2	11	6	4	1	3	2	1	0	6	2	1	1
**23**	Golden Hamster (Mesocricetus auratus)	24	1	1	6	3	8	3	2	0	4	2	1	0	1	1	1	1
**24**	Prairie vole (Microtus ochrogaster)	25	2	2	4	1	8	4	4	2	1	0	0	0	2	2	1	1
**25**	Northern American deer	26	1	1	4	2	11	6	2	0	0	0	0	0	3	2	1	1

	mouse (Peromyscus maniculatus bairdii)																	
**26**	Mouse (Mus musculus)	27	1	1	7	5	10	5	2	0	1	1	1	0	2	1	2	2
**27**	Algerian mouse (Mus spretus)	28	1	1	6	4	9	5	2	0	1	1	1	0	2	1	2	2
**28**	Ryukyu mouse (Mus caroli)	29	1	1	7	4	7	5	2	0	1	1	1	0	2	1	2	2
**29**	Shrew mouse (Mus pahari)	30	1	1	7	4	10	5	2	1	2	2	2	0	2	1	2	2
**30**	Rat (Rattus norvegicus)	31	1	1	6	4	11	4	3	0	1	0	2	0	6	2	1	1
**31**	Upper Galilee mountains blind mole rat (Nannospalax galili)	32	1	1	6	3	7	5	1	0	3	1	1	0	2	2	3	3
**32**	Lesser Egyptian jerboa (Jaculus jaculus)	33	1	nr.	3	nr.	5	nr.	0	nr.	0	nr.	0	nr.	1	nr.	3	nr.
**33**	Rabbit (Oryctolagus cuniculus)	34	2	1	3	2	5	4	10	2	4	0	2	0	5	2	4	2
**34**	Ferret (Mustela putorius furo)	35	1	1	3	2	5	5	2	1	4	2	1	1	2	2	3	3
**35**	Dog (Canis familiaris)	36	1	nr.	1	nr.	2	nr.	0	nr.	4	nr.	0	nr.	1	nr.	2	nr.
**36**	Cat (Felis catus)	37	1	1	6	1	13	10	3	1	1	0	2	0	4	1	6	4
**37**	Horse (Equus caballus)	38	1	1	7	2	7	4	2	1	2	2	0	0	3	2	5	3
**38**	Cow (Bos taurus)	39	10	1	13	3	21	6	15	0	4	1	7	0	4	2	14	3
**39**	Sheep(Ovis aries)	40	1	1	6	3	6	6	2	0	1	1	0	0	2	2	3	3
**40**	Armadillo (Dasypus novemcinctus)	41	1	1	7	1	7	4	3	1	6	4	2	0	5	2	4	3
**41**	Tasmanian devil (Sarcophilus harrisii)	42	2	nr.	3	nr.	7	nr.	0	nr.	0	nr.	0	nr.	1	nr.	3	nr.
**42**	Opossum (Monodelphis domestica)	43	2	1	8	5	11	7	6	1	1	1	4	0	6	1	5	3
**43**	Platypus (Ornithorhynchu s anatinus)	44	4	nr.	3	nr.	9	nr.	1	nr.	0	nr.	0	nr.	3	nr.	4	nr.
**44**	Chicken (Gallus gallus)	45	1	1	5	1	7	5	3	3	2	2	0	0	2	2	2	1
**45**	Duck (Anas platyrhynchos)	46	1	nr.	0	nr.	6	nr.	2	nr.	0	nr.	0	nr.	1	nr.	1	nr.
**46**	Flycatcher (Ficedula albicollis)	47	1	nr.	0	nr.	7	nr.	1	nr.	0	nr.	1	nr.	1	nr.	1	nr.
**47**	Anole lizard (Anolis carolinensis)	48	1	1	4	2	9	5	4	2	1	0	1	1	3	0	4	3
**48**	Coelacanth (Latimeria chalumnae)	49	2	2	3	1	7	4	1	0	0	0	3	0	7	1	1	1
**49**	Platyfish (Xiphophorus maculatus)	50	1	0	0	0	6	1	8	3	3	1	3	1	4	0	7	2
**50**	Medaka (Oryzias latipes)	51	1	0	3	2	1	0	2	2	1	0	1	0	0	0	3	2
**51**	Tilapia (Oreochromis niloticus)	52	0	nr.	2	nr.	1	nr.	1	nr.	1	nr.	0	nr.	0	nr.	2	nr.
**52**	Tetraodon (Tetraodon nigroviridis)	53	0	0	2	2	3	1	2	1	1	1	2	0	0	0	3	2
**53**	Zebrafish (Danio rerio)	54	1	0	2	2	2	1	0	0	1	1	2	1	7	2	1	0
**51**	Tilapia (Oreochromis niloticus)	55	0	nr.	4	nr.	4	nr.	6	nr.	0	nr.	1	nr.	1	nr.	4	nr.
**54**	Cave fish (Astyanax mexicanus)	56	7	nr.	14	nr.	18	nr.	14	nr.	4	nr.	8	nr.	10	nr.	20	nr.
**53**	Zebrafish (Danio rerio)	57	3	0	2	1	4	1	2	1	3	0	0	0	4	0	4	3
**53**	Zebrafish (Danio rerio)	58	4	nr.	3	nr.	13	nr.	8	nr.	3	nr.	4	nr.	3	nr.	10	nr.
**55**	Spotted gar (Lepisosteus oculatus)	59	5	0	5	1	12	3	12	0	5	0	7	0	16	0	8	2
	**Primates (N=17)**	**M. :**	**1**	**1**	**6**	**3**	**10**	**7**	**5**	**0**	**3**	**2**	**3**	**0**	**4**	**1**	**5**	**3**

**Rodents (N=16)**	**M.:**	**1**	**1**	**6**	**3**	**8**	**5**	**2**	**1**	**2**	**1**	**1**	**0**	**2**	**2**	**2**	**2**
**Placental mammals (N=7)**	**M.:**	**1**	**1**	**6**	**2**	**7**	**6**	**2**	**1**	**4**	**2**	**1**	**0**	**3**	**2**	**4**	**3**
**Marsupials and Platypus (N=3)**	**M.:**	**2**	**1**	**3**	**5**	**9**	**7**	**1**	**1**	**0**	**1**	**0**	**0**	**3**	**1**	**4**	**3**
**Reptiles and Birds and Coelacanth (N=5)**	**M.:**	**1**	**1**	**3**	**1**	**7**	**5**	**2**	**2**	**0**	**0**	**1**	**0**	**2**	**1**	**1**	**1**
**Ray finned fishes - abca1a (N=6)**	**M.:**	**1**	**0**	**2**	**2**	**3**	**1**	**2**	**1**	**1**	**1**	**2**	**0**	**2**	**0**	**3**	**2**
**Ray finned fishes abca1b (N=3)**	**M.:**	**4**	**0**	**4**	**1**	**9**	**1**	**7**	**1**	**3**	**0**	**3**	**0**	**4**	**0**	**7**	**3**

Abbreviations: M., median; nr., not relevant; sp., species; tr., transcript; Wh., whole; Af., after; In., intron;

Supplemental Table S4: Upstream ATG and non-ATG start as well as stop codon comparison – number and position.

Supplemental Table S5

Table S5: 5'UTR GC content versus length comparison

Species	GC content (%)				Whole 5'UTR length (nts)
	Before In. 1	After In. 1	Whole r.	Subtr. After_In._1 - Before_In._1	
Human	58.7	67.4	60.8	8.7	395
Macaque	58.1	64.1	59.3	6	452
Mouse Lemur	61.9	62	61.9	0.1	402
Rabbit	57.9	61.1	58.6	3.2	418
Mouse	63.2	64	63.4	0.8	320
Squirrel	60	60.4	60.1	0.4	371
Cat	60	61.6	60.4	1.6	399
Armadillo	63.7	67	64.5	3.3	400
Tasmanian devil	n. r.	n. r.	57.9	n. r.	95
Opossum	51.7	61.1	53.8	9.4	424
Platypus	n. r.	n. r.	52.6	n. r.	137
Chicken	77.9	49.3	67.5	-28.6	206
Flycatcher	n. r.	n. r.	46.1	n. r.	76
Anole lizard	63.6	49.4	59.9	-14.2	294
Coelacanth	32	47.3	34.8	15.3	296
Zebrafish (A1a)	37.8	52.5	41	14.7	188
Spotted gar (A1a)	42.5	60	43.6	17.5	637

Abbreviations: In, Intron; n. r., not relevant; r., region; subtr., subtraction;

Supplemental Table S5: GC content versus length comparison in 5'UTRs of orthologous *ABCA1* genes.

Supplemental Table S6

Table S6: Analysis of conserved RNA motifs and sites in human ABCA1 5'UTR - detailed information

		Human		
**Motif_Type**	**Motif_Name**	**Position**	**Length**	**Sequence**
exon splicing enhancer (ESE)	ct/cgrp intron downstream from exon 4	217 ~ 225	9	tttttcccc
	sc35 - exonic splicing enhancer	113 ~ 120	8	gacccgca
	sc35 - exonic splicing enhancer	356 ~ 363	8	ggctcccg
exon splicing silencer (ESS)	fibronectin eda exon	260 ~ 265	6	caaggg
intron splicing enhancer (ISE)	gh-1 intron 3	384 ~ 393	10	ggctgaggga
	gh-1 intron 3	105 ~ 114	10	ggggccggga
	ighg2 cgamma2 (immunoglobulin heavy chain subclass g2 - cgamma2 gene) - intron 1	99 ~ 104	6	gtgagt
	ighg2 cgamma2 (immunoglobulin heavy chain subclass g2 - cgamma2 gene) - intron 1	331 ~ 336	6	gtgagc
	ctnt, exon 5	253 ~ 259	7	ggcttgt
	ctnt, exon 5	384 ~ 390	7	ggctgag
rho-independent terminator	Rho-independent Terminator	197 ~ 215	19	cagggcgctttgctccttg
Transcriptional regulatory motif (TRM)	Sp1	158 ~ 170	13	gcagggcggggag
	Elk-1	243 ~ 256	14	ttctccggaaggct
	TATA	51 ~ 65	15	ctataaaaggaacta
	Msx-1	81 ~ 89	9	ccgtaattg
	TFIIA	52 ~ 63	12	tataaaaggaac
	Kid3	315 ~ 319	5	ccacg
	NFAT1	295 ~ 300	6	ggaaaa
	NF-AT4	295 ~ 300	6	ggaaaa
	HOXA13	53 ~ 58	6	ataaaa
	ER81	246 ~ 255	10	tccggaaggc
	PEA3	246 ~ 255	10	tccggaaggc
	ETV7	246 ~ 255	10	tccggaaggc
	E2F-3	162 ~ 168	7	ggcgggg
UTR motifs	Musashi binding element (MBE)	20 ~ 24	5	atagt
microRNA target sites	hsa-miR-4435	323 ~ 345	23	tccctgctgtgagctctggccgc
	hsa-miR-5581-5p	236 ~ 256	21	tctccccttctccggaaggct

Supplemental Table S6: Analysis of conserved RNA motifs and sites in human ABCA1 5'UTR – detailed information.

Supplementary Information 2 – Supplementary Figures

Supplemental Figure S1

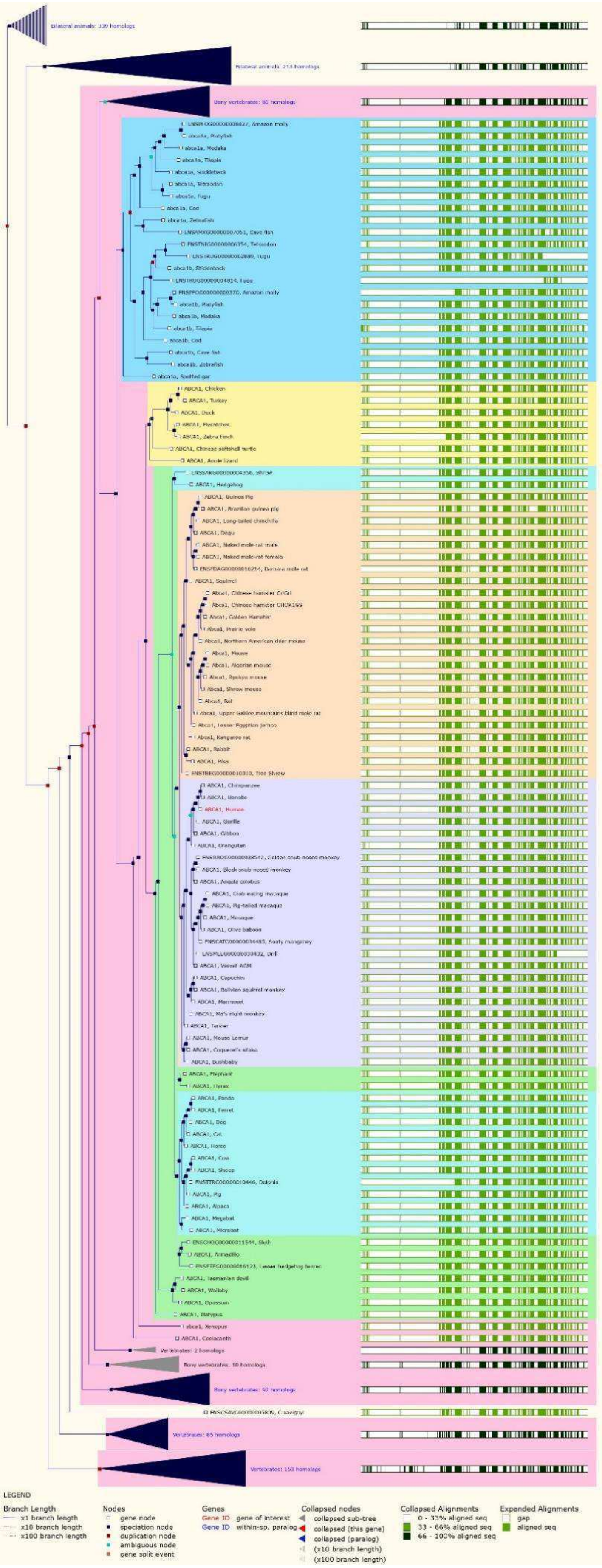

Supplemental Figure S1: The *ABCA1* gene tree created by the Gene Tree tool – Ensembl database.

Supplemental Figure S2

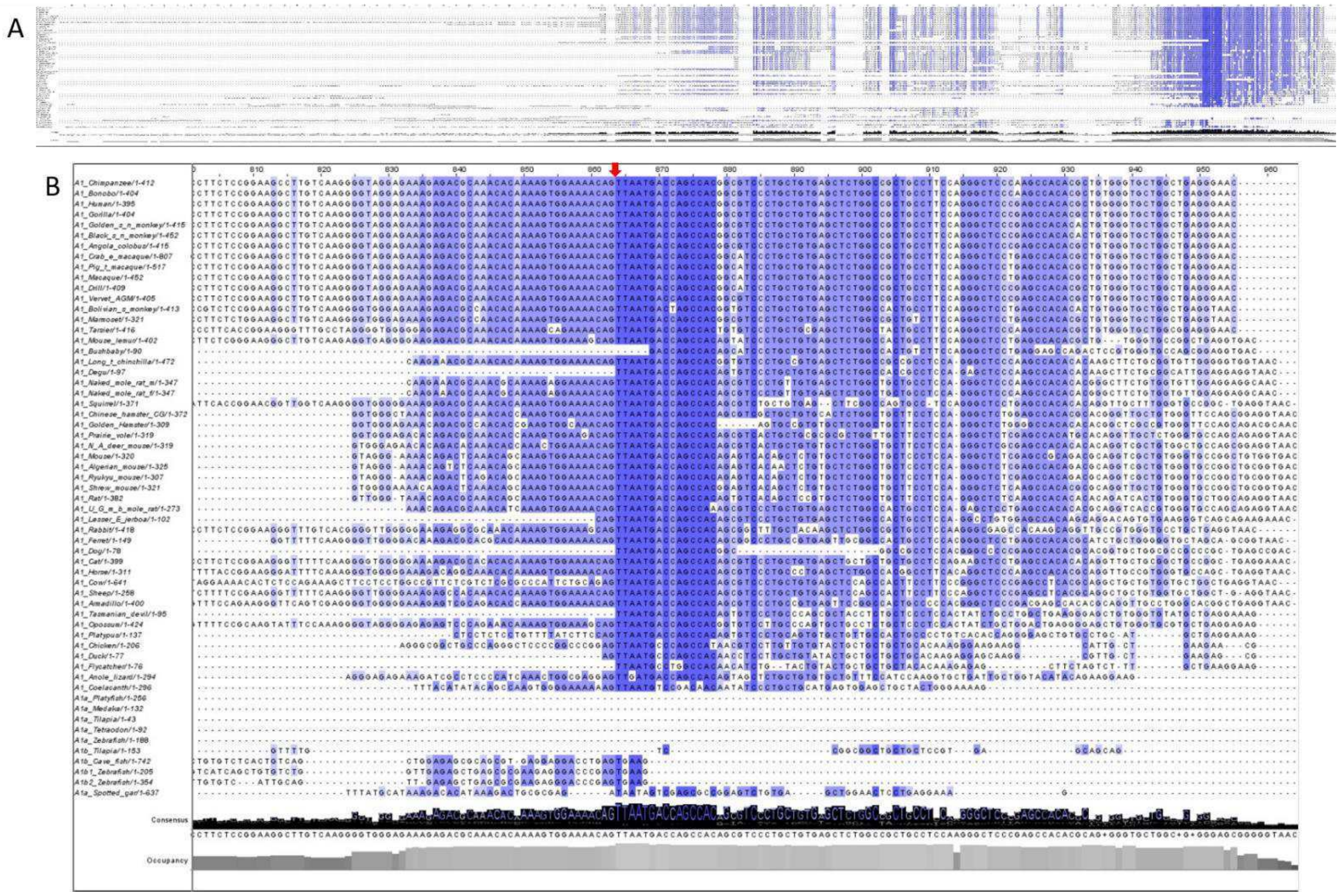

Supplemental Figure S2: Results of the ABCA1 5’UTR multi-sequence alignment analysis in the ClustalO program performed on 59 transcripts downloaded from the Ensembl database. Lines represent transcripts, which were aligned according to the ClustalO algorithm; the conservation of nucleotides among vertebrate species is highlighted with the most conserved ones having the darkest color. A) Alignment of the whole 5’UTRs. 5’UTR segments, which contain conserved nucleotides can be distinguished. B) Detail of the 5’UTR segment containing the most conserved sequences; the position of the spliced Intron 1 is marked by a red arrow.

Supplemental Figure S3

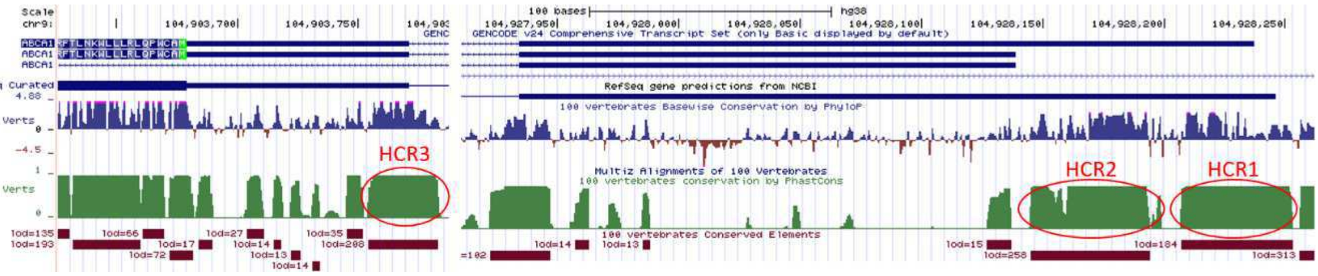

Supplemental Figure S3: Results of the conservation track focused on the 5’UTR region of the human *ABCA1* gene performed in UCSC Genome Browser. Three highly conserved regions (HCR1-HCR3) can be distinguished. HCR3 is overlapping with the most conserved sequence displayed on Figure 4 in the main text.

Supplemental Figure S4

↓ specifies the position of Intron 1

CTG uCTGs are marked with red color

>A1_Chimpanzee

[illegible]

>A1_Bonobo

[illegible]

>A1_Human

[illegible]

>A1_Gorilla

[illegible]

>A1_Golden_s_n_monkey

[illegible]

>A1_Black_s_n_monkey

[illegible]

>A1_Angola_colobus

[illegible]

>A1_Crab_e_macaque

[illegible]

>A1_Pig_t_macaque

[illegible]

>A1_Macaque

[illegible]

>A1_Drill

[illegible]

>A1_Vervet_AGM

[illegible]

>A1_Bolivian_s_monkey

[illegible]

>A1_Marmoset

[illegible]

>A1_Tarsier

[illegible]

>A1_Mouse_lemur

[illegible]

>A1_Bushbaby_no intron

[illegible]

>A1_Long_t_chinchilla

[illegible]

>A1_Degu_no intron

[illegible]

>A1_Naked_mole_rat_m

[illegible]

>A1_Naked_mole_rat_f

[illegible]

>A1_Squirrel

[illegible]

>A1_Chinese_hamster_CG

[illegible]

>A1_Golden_Hamster

[illegible]

>A1_Prairie_vole

[illegible]

>A1_N_A_deer_mouse

[illegible]

>A1_Mouse

[illegible]

>A1_Algerian_mouse

[illegible]

>A1_Ryukyu_mouse

[illegible]

>A1_Shrew_mouse

[illegible]

>A1_Rat

[illegible]

>A1_U_G_m_b_mole_rat

[illegible]

>A1_Lesser_E_jerboa_no intron

[illegible]

>A1_Rabbit

[illegible]

>A1_Ferret

[illegible]

>A1_Dog_no intron

[illegible]

>A1_Cat

[illegible]

>A1_Horse

[illegible]

>A1_Cow

[illegible]

>A1_Sheep

[illegible]

>A1_Armadillo

[illegible]

>A1_Tasmanian_devil_no intron

[illegible]

>A1_Opossum

[illegible]

>A1_Platypus_no intron

[illegible]

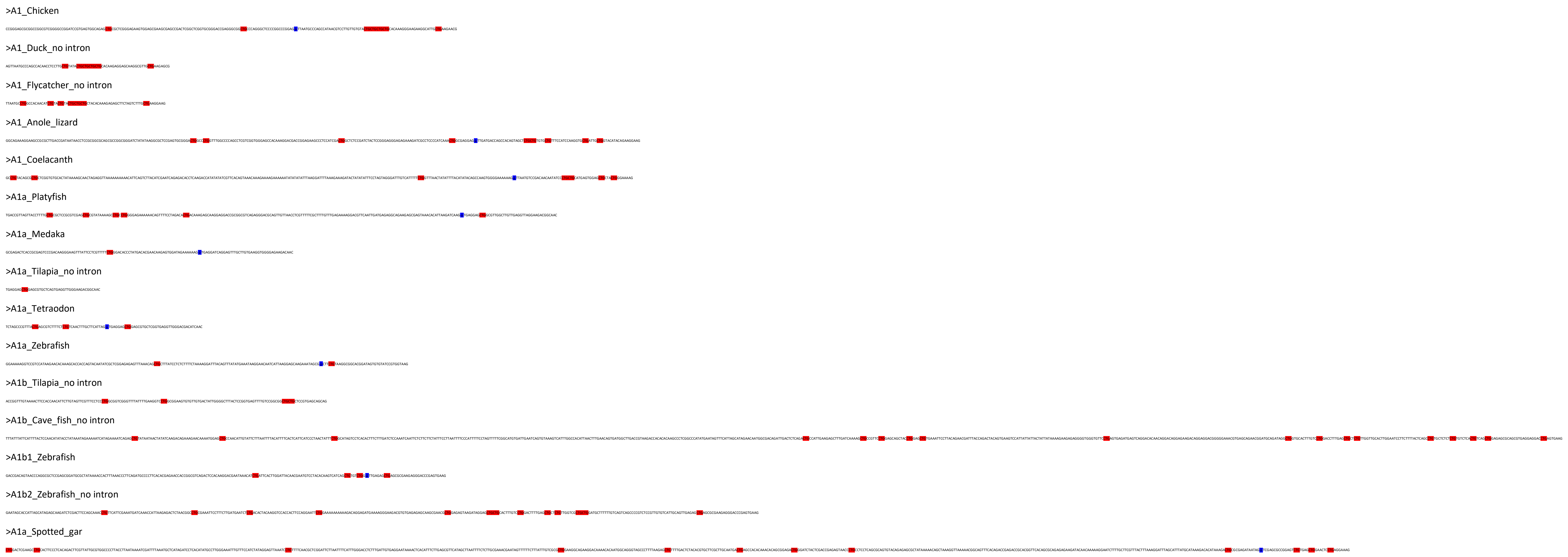

Supplemental Figure S4: The analysis of uCTG distribution within 5'UTRs of *ABCA1* genes from 55 species, 5'UTR sequences were downloaded from the Ensembl database.

Supplemental Figure S5

↓ specifies the position of Intron 1

TTG uTTGs are marked with red color

>A1_Chimpanzee

[illegible]

>A1_Bonobo

[illegible]

>A1_Human

[illegible]

>A1_Gorilla

[illegible]

>A1_Golden_s_n_monkey

[illegible]

>A1_Black_s_n_monkey

[illegible]

>A1_Angola_colobus

[illegible]

>A1_Crab_e_macaque

[illegible]

>A1_Pig_t_macaque

[illegible]

>A1_Macaque

[illegible]

>A1_Drill

[illegible]

>A1_Vervet_AGM

[illegible]

>A1_Bolivian_s_monkey

[illegible]

>A1_Marmoset

[illegible]

>A1_Tarsier

[illegible]

>A1_Mouse_lemur

[illegible]

>A1_Bushbaby_no intron

[illegible]

>A1_Long_t_chinchilla

[illegible]

>A1_Degu_no intron

[illegible]

>A1_Naked_mole_rat_m

[illegible]

>A1_Naked_mole_rat_f

[illegible]

>A1_Squirrel

[illegible]

>A1_Chinese_hamster_CG

[illegible]

>A1_Golden_Hamster

[illegible]

>A1_Prairie_vole

[illegible]

>A1_N_A_deer_mouse

[illegible]

>A1_Mouse

[illegible]

>A1_Algerian_mouse

[illegible]

>A1_Ryukyu_mouse

[illegible]

>A1_Shrew_mouse

[illegible]

>A1_Rat

[illegible]

>A1_U_G_m_b_mole_rat

[illegible]

>A1_Lesser_E_jerboa_no intron

[illegible]

>A1_Rabbit

[illegible]

>A1_Ferret

[illegible]

>A1_Dog_no intron

[illegible]

>A1_Cat

[illegible]

>A1_Horse

[illegible]

>A1_Cow

[illegible]

>A1_Sheep

[illegible]

>A1_Armadillo

[illegible]

>A1_Tasmanian_devil_no intron

[illegible]

>A1_Opossum

[illegible]

>A1_Platypus_no intron

[illegible]

>A1_Chicken

CCGGGAGCGCGGCCGGCGTCGGGGCCGGATCCGTGAGTGGCAGAGCTGCGCTCGGGAGAAGTGGAGCGAAGCGAGCCGACTCGGCTCGGTGCGGGACCGAGGGCGGCTGCCCAGGGCTCCCCGGCCCGGAGTTAATGCCCAGCCATAACGTCCTTGTTGTGTACTGCTGCTGCTGCACAAAGGGAAGAAGGCATTGCTGAAGAACG

>A1_Duck_no intron

AGTTAATGCCCAGCCACAACCTCCTTGCTGTATACTGCTGCTGCTGCACAAGAGGAGCAAGGCGTTGCTGAAGAGCG

>A1_Flycatcher_no intron

TTAATGCCTGGCCACAACATCTGTACTGTACTGCTGCTGCTACACAAAGAGAGCTTCTAGTCTTTGCTGAAGGAAG

>A1_Anole_lizard

GGCAGAAAGGAAGCCGCGCTTGACCGATAATAACCTCCGCGGCGCAGCGCCGGCGGGATCTATATAAGGCGCTCCGAGTGCGGGACTGGCCCTGGTTTGGCCCCAGCCTCGTCGGTGGGAGCCACAAAGGACGACCGGAGAAGCCCTCCATCGACTGGCTCTCCGATCTACTCCGGGAGGGAGAGAAAGATCGCCTCCCCATCAAACTGGCGAGGAGTTGATGACCAGCCACAGTAGCTCTGCTGTGTGCTGTTTCCATCCAAGGTGCTGATTGCTGGTACATACAGAAGGAAG

>A1_Coelacanth

GCCTGTACAGCGCTGCTCGGTGTGCACTATAAAAGCAACTAGAGGTTAAAAAAAAAACATTCAGTCTTACATCGAATCAGAGACACCTCAAGACCATATATATCGTTCACAGTAAACAAAGAAAAGAAAAAATATATATATTTAAGGATTTTAAAGAAAGATACTATATATTTCCTAGTAGGGATTTGTCATTTTTCTGGTTTAACTATATTTTACATATACAGCCAAGTGGGGAAAAAAGTTAATGTCCGACAACAATATCCCTGCTGCATGAGTGGAGCTGCTACTGGGAAAAG

>A1a_Platyfish

TGACCGTTAGTTACCTTTTGCTGCGCTCCGCGTCGAGCTGCGTATAAAAGCCTGCCTGGGGAGAAAAAACAGTTTTCCTAGACACTGACAAAGAGCAAGGAGGACCGCGGCGTCAGAGGGACGCAGTTGTTAACCTCGTTTTTCGCTTTTGTTTGAGAAAAGGACGTTCAATTGATGAGAGGCAGAAGAGCGAGTAAACACATTAAGATCAAGTGAGGAGCTGGCGTTGGCTTGTTGAGGTTAGGAAGACGGCAAC

>A1a_Medaka

GCGAGACTCACCGCGAGTCCCGACAAGGGAAGTTTATTCCTCGTTTTTCTGGGACACCCTATGACACGAACAAGAGTGGATAGAAAAAAGTGAGGATCAGGAGTTTGCTTGTGAAGGTGGGGAGAAGACAAC

>A1a_Tilapia_no intron

TGAGGAGCTGGAGCGTGCTCAGTGAGGTTGGGAAGACGGCAAC

>A1a_Tetraodon

TCTAGCCCGTTTACTGAGCGTCTTTTCTCTGTCAACTTTGCTTCATTAGTGAGGAGCTGGAGCGTGCTCGGTGAGGTTGGGACGACATCAAC

>A1a_Zebrafish

GGAAAAAGGTCCGTCCATAAGAACACAAAGCACCACCAGTACAATATCGCTCGGAGAGAGTTTAAACAGCTGCTTTATCCTCTCTTTTCTAAAAGGATTTACAGTTTATATGAAATAAGGAACAATCATTAAGGAGCAAGAAATAGCGCTTCTGTAAGGCGGCACGGATAGTGTGTATCCGTGGTAAG

>A1b_Tilapia_no intron

ACCGGTTTGTAAAACTTCCACCAACATTCTTGTAGTTCGTTTCCTCCCTGGCGGTCGGGTTTTATTTTGAAGGTCCTGGCGGAAGTGTGTTGTGACTATTGGGGCTTTACTCCGGTGAGTTTTGTCCGGCGGCTGCTGCTCCGTGAGCAGCAG

>A1b_Cave_fish_no intron

TTTATTTATTCATTTTACTCCAACATATACCTATAAATAGAAAAATCATAGAAAATCAGAGCTGTATAATAACTATATCAAGACAGAAAGAACAAAATGGAGCTGCCAACATTGTATTCTTTAATTTTACATTTTCACTCATTCATCCCTAACTATTTCTGGCATAGTCCTCACACTTTCTTTGATCTCCAAATCAATTCTCTTCTTCTATTTCCTTAATTTTCCCATTTTTCCTAGTTTTTCGGCATGTGATTGAATCAGTGTAAAGTCATTTGGCCACATTAACTTTGAACAGTGATGGCTTGACCGTAAGACCACACACAAGCCCTCGGCCCATATGAATAGTTTCATTAGCATAGAACAATGGCGACAGATTGACTCTCAGACTGCCATTGAAGAGCTTTGATCAAAAGCTGCCGTTCCTGGAGCAGCTACCTGGAGCTGTGAAATTCCTTACAGAACGATTTACCAGACTACAGTGAAGTCCATTATTATTACTATTATAAAAGAAGAGAGGGGTGGGTGTTCCTGAGTGAGATGAGTCAGGACACAACAGGACAGGAGAAGACAGGAGGACGGGGGAAACGTGAGCAGAACGGATGCAGATAGGCTGGTGCACTTTGTCCTGGACCTTTGAGCTGCTCTGTTGGTTGCACTTGGAATCCTTCTTTTACTCAGCCTGTGCTCTCTCTGTGTCTCACTGTCAGCTGGAGAGCGCAGCGTGAGGAGGACCTGAGTGAAG

>A1b1_Zebrafish

GACCGACAGTAACCCAGGCGCTCCGAGCGGATGCGCTATAAAACCACTTTAAACCCTTCAGATGCCCCTTCACACGAGAACCACCGGCGTCAGACTCCACAAGGACGAATAAACATCTGATTCACTTGGATTACAACGAATGTCCTACACAAGTCATCAGCTGTGTCTGGTTGAGAGCTGAGCGCGAAGAGGGACCCGAGTGAAG

>A1b2_Zebrafish_no intron

GAATAGCACCATTAGCATAGAGCAAGATCTCGACTTCCAGCAAACCTGTTCATTCGAAATGATCAAACCATTAAGAGACTCTAACGGCCTGCGAAATTCCTTTCTTGATGAATCTCTGACACTACAAGGTCCACCACTTCCAGGAATTCTGGAAAAAAAAAAGACAGGAGATGAAAAGGGAAGACGTGTGAGAGAGCAAGCGAACGCTGGAGAGTAAGATAGGAGCTGCTGCACTTTGTCCTGGACTTTGAGCTGCTCTGTTGGTCGCTGCTGGATGCTTTTTGTCAGTCAGCCCCGTCTCCGTTGTGTCATTGCAGTTGAGAGCTGAGCGCGAAGAGGGACCCGAGTGAAG

>A1a_Spotted_gar

CTGGACTCGAAGCCTGCACTTCCCTCACAGACTTCGTTATTGCGTGGCCCCTTACCTTAATAAAATCGATTTTAAATGCTCATAGATCCTCACATATGCCTTGGGAAATTTGTTTCCATCTATAGGAGTTAAATCCTGTTTTCAACGCTCGGATTCTTAATTTTCATTTGGGACCTCTTTGATTGTGAGGAATAAAACTCACATTTCTTGAGCGTTCATAGCTTAATTTTCTCTTGCGAAACGAATAGTTTTTTCTTTATTTGTCGCGCTGGAAGGCAGAAGGACAAAACACAATGGCAGGGTAGCCCTTTTAAGAGCTGTTTTGACTCTACACGTGCTTCGCTTGCAATGACTGAGCCACACAAACACAGCGGAGACTGGGATCTACTCGACCGAGAGTAACCCTGCCTCCTCAGCGCAGTGTACAGAGAGCGCTATAAAAACAGCTAAAGGTTAAAAACGGCAGTTTCACAGACCGAGACCGCACGGTTCACAGCGCAGAGAGAAGATACAACAAAAAGGAATCTTTTGCTTCGTTTACTTTAAAGGATTTAGCATTTATGCATAAAGACACATAAAGACTGCGCGAGATAATAGTCGAGCGCCGGAGTCTGTGAGCTGGAACTCCTGAGGAAAG

Supplemental Figure S5: The analysis of uTTG distribution within 5’UTRs of *ABCA1* genes from 55 species, 5’UTR sequences were downloaded from the Ensembl database.

Supplemental Figure S6

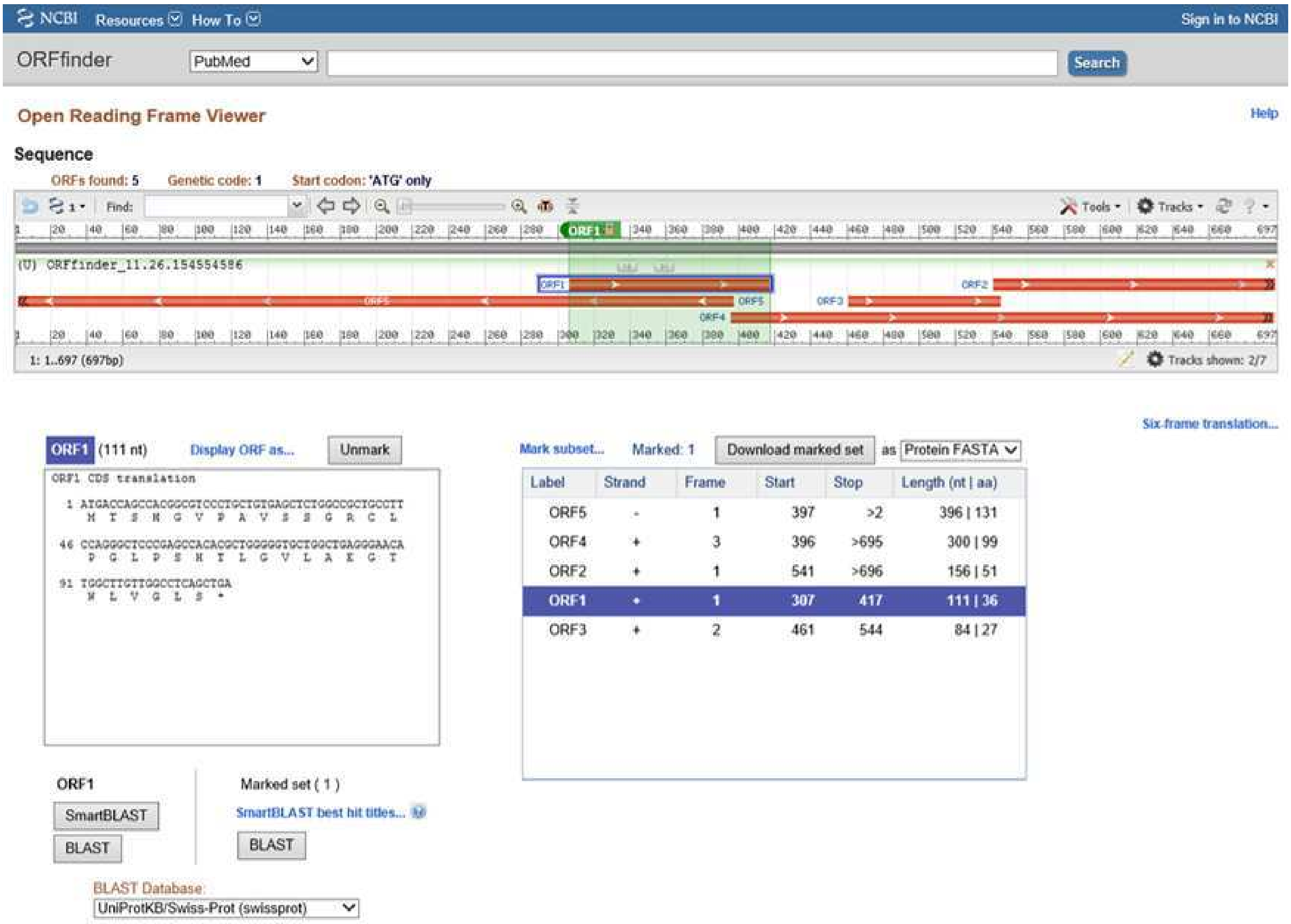

Supplemental Figure S6: ORF prediction on the 5' end of human *ABCA1* transcript calculated by ORFfinder. Upstream ORF discussed within the main text was predicted as ORF1 and known main ORF as ORF4.

Supplemental Figure S7

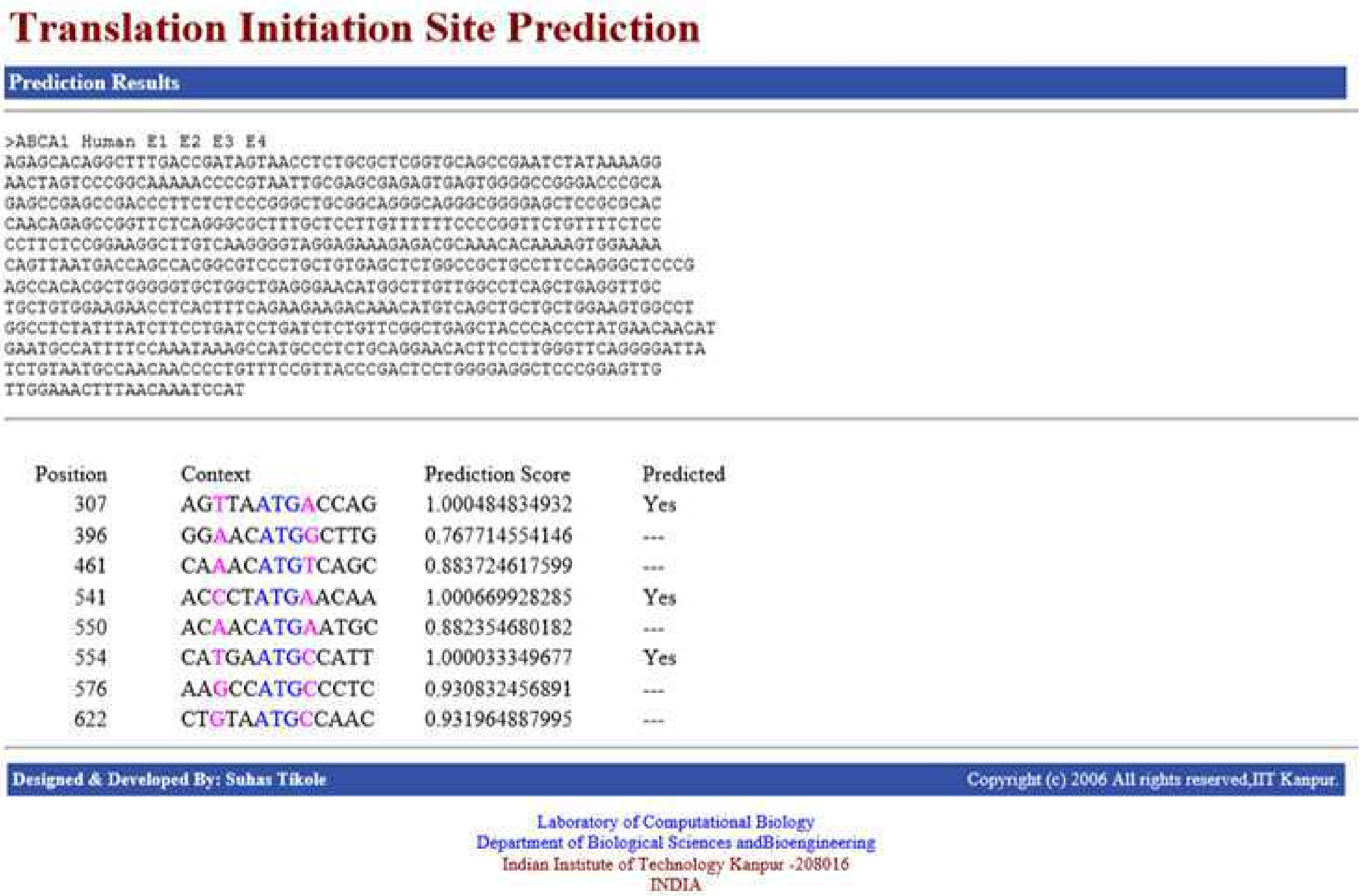

Translation Initiation Site Prediction

Prediction Results

```
>ABCA1 Human E1 E2 E3 E4
AGAGCACAGGCTTTGACCGATAGTAACCTCTGCGCTCGGTGCAGCCGAATCTATAAAAGG
AACTAGTCCCGGCAAAAACCCCGTAATTGCGAGCGAGAGTGAGTGGGGCCGGGACCCGCA
GAGCCGAGCCGACCCTTCTCTCCCGGGCTGCGGCAGGGCAGGGCGGGGAGCTCCGCGCAC
CAACAGAGCCGGTTCTCAGGGCGCTTTGCTCCTTGTTTTTTCCCCGGTTCTGTTTTCTCC
CCTTCTCCGGAAGGCTTGTCAAGGGGTAGGAGAAAGAGACGCAAACACAAAAGTGGAAAA
CAGTTAATGACCAGCCACGGCGTCCCTGCTGTGAGCTCTGGCCGCTGCCTTCCAGGGCTCCCG
AGCCACACGCTGGGGGTGCTGGCTGAGGGAACATGGCTTGTTGGCCTCAGCTGAGGTTGC
TGCTGTGGAAGAACCTCACTTTCAGAAGAAGACAAACATGTCAGCTGCTGCTGGAAGTGGCCT
GGCCTCTATTTATCTTCCTGATCCTGATCTCTGTTCGGCTGAGCTACCCACCCTATGAACAACAT
GAATGCCATTTTCCAAATAAAGCCATGCCCTCTGCAGGAACACTTCCTTGGGTTCAGGGGATTA
TCTGTAATGCCAACAACCCCTGTTTCCGTTACCCGACTCCTGGGGAGGCTCCCGGAGTTG
TTGGAAACTTTAACAAATCCAT
```

Position	Context	Prediction Score	Predicted
307	AGTTAATGACCAG	1.000484834932	Yes
396	GGAACATGGCTTG	0.767714554146	---
461	CAAACATGTCAGC	0.883724617599	---
541	ACCCTATGAACAA	1.000669928285	Yes
550	ACAACATGAATGC	0.882354680182	---
554	CATGAATGCCATT	1.000033349677	Yes
576	AAGCCATGCCCTC	0.930832456891	---
622	CTGTAATGCCAAC	0.931964887995	---

Designed & Developed By: Suhas Tikole

Copyright (c) 2006 All rights reserved,IIT Kanpur.

Laboratory of Computational Biology
Department of Biological Sciences andBioengineering
Indian Institute of Technology Kanpur -208016
INDIA

Supplemental Figure S7: Results obtained from the WeakAUG server.

Supplemental Figure S8

Color marking of base repetitions: AA/AAA, TT/TTT, CC/CCC, GG/GGG

↓ specifies the position of Intron 1

>A1_Chimpanzee

[illegible]

>A1_Bonobo

[illegible]

>A1_Human

[illegible]

>A1_Gorilla

[illegible]

>A1_Golden_s_n_monkey

[illegible]

>A1_Black_s_n_monkey

[illegible]

>A1_Angola_colobus

[illegible]

>A1_Crab_e_macaque

[illegible]

>A1_Pig_t_macaque

[illegible]

>A1_Macaque

[illegible]

>A1_Drill

[illegible]

>A1_Vervet_AGM

[illegible]

>A1_Bolivian_s_monkey

[illegible]

>A1_Marmoset

[illegible]

>A1_Tarsier

[illegible]

>A1_Mouse_lemur

[illegible]

>A1_Bushbaby_no intron

[illegible]

>A1_Long_t_chinchilla

[illegible]

>A1_Degu_no intron

[illegible]

>A1_Naked_mole_rat_m

[illegible]

>A1_Naked_mole_rat_f

[illegible]

>A1_Squirrel

[illegible]

>A1_Chinese_hamster_CG

[illegible]

>A1_Golden_Hamster

[illegible]

>A1_Prairie_vole

[illegible]

>A1_N_A_deer_mouse

[illegible]

>A1_Mouse

[illegible]

>A1_Algerian_mouse

[illegible]

>A1_Ryukyu_mouse

[illegible]

>A1_Shrew_mouse

[illegible]

>A1_Rat

[illegible]

>A1_U_G_m_b_mole_rat

[illegible]

>A1_Lesser_E_jerboa_no intron

[illegible]

>A1_Rabbit

[illegible]

>A1_Ferret

[illegible]

>A1_Dog_no intron

[illegible]

>A1_Cat

[illegible]

>A1_Horse

[illegible]

>A1_Cow

[illegible]

>A1_Sheep

[illegible]

>A1_Armadillo

[illegible]

>A1_Tasmanian_devil_no intron

[illegible]

>A1_Opossum

[illegible]

>A1_Platypus_no intron

[illegible]

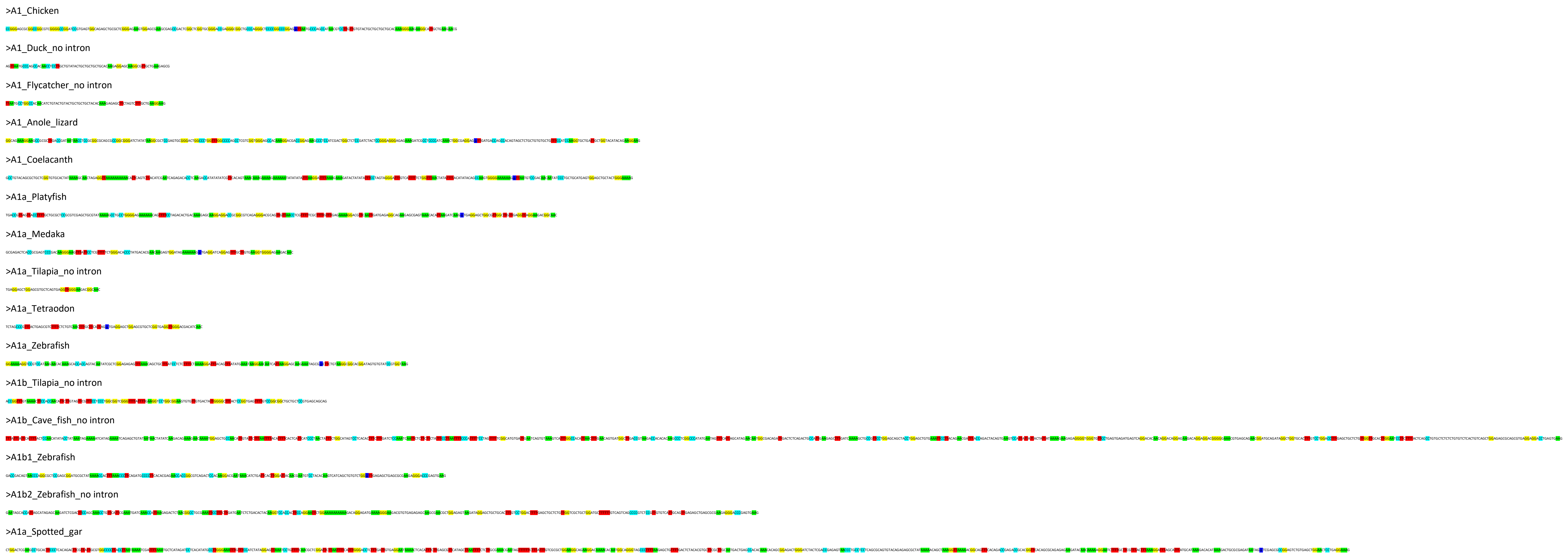

Supplemental Figure S8: Analysis of specific base-repetition-rich subregions in 5’UTRs of orthologous *ABCA1* genes, 5’UTR sequences of 55 species were downloaded from the Ensembl database.

Supplemental Figure S9

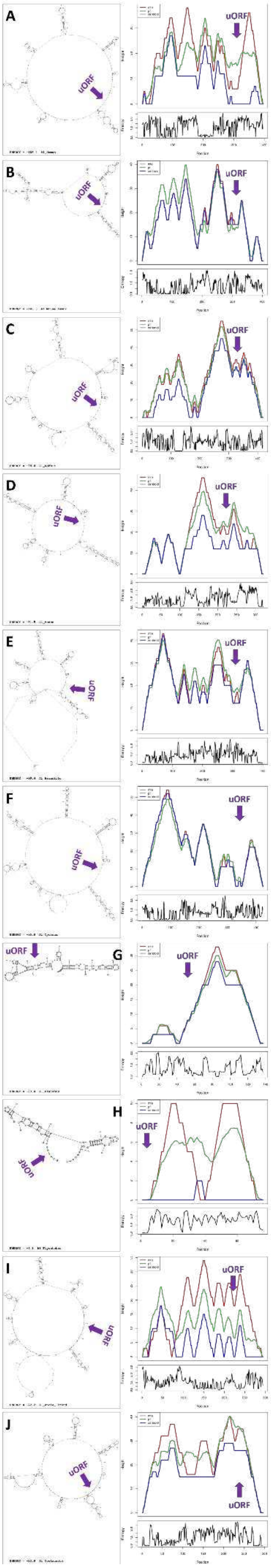

Supplemental Figure S9: Secondary structure comparison of *ABCA1* 5'UTRs in 10 vertebrate species. Two web servers – RNAstructure (secondary structure diagrams) and RNAfold (mountain plots) were employed independently. Results of the following species are disclosed: A) Human, B) Mouse lemur, C) Rabbit, D) Mouse, E) Armadillo, F) Opossum, G) Platypus, H) Flycatcher, I) Anole lizard, and J) Coelacanth. Start position of the uORF discussed in the main text is marked with an arrow.

Supplemental Figure S10

Gene: ABCA1

Human (GRCh37.p13)

Transcript: ABCA1-002 ENST00000374736.3

5'UTR sequence

TATA TFIIA HOXA13

AGAGCACAGGCTTTGACCGATAGTAACCTCTGCGCTCGGTGCAGCCGAATCTATAAAAGG

Ighg2 Gh-1 Sc35

AACTAGTCCCGGCAAAAACCCCGTAATTGCGAGCGAGAGTGAGTGGGGCCGGGACCCGCA

GAGCCGAGCCGACCCTTCTCTCCCGGGCTGCGGCAGGGCAGGGCGGGGAGCTCCGCGCAC

CAACAGAGCCGGTTCTCAGGGCGCTTTGCTCCTTGTTTTTTCCCCGGTTCTGTTTTCTCC

Ctnt FEE NFAT1/4

CCTTCTCCGGAAGGCTTGTCAAGGGGTAGGAGAAAGAGACGCAAACACAAAAGTGGAAAA

uORF Ighg2 Sc35

CAGTTAATGACCAGCCACGGCGTCCCTGCTGTGAGCTCTGGCCGCTGCCTTCCAGGGCTCCCG

Ctnt Gh-1

AGCCACACGCTGGGGGTGCTGGCTGAGGGAAC

Notes:

5'UTR variants according to Ensembl in blue color

5'UTR hot spots defined by the study in red

Nucleotides matching both criteria in orange

Supplemental Figure S10: Analysis of the human *ABCA1* 5'UTR variants from Ensembl versus hot spots defined by the study.
